# Novel Metabolites from the Marine-Derived Fungus *Peniophora* sp. SCSIO41203 Show Promising In Vitro Antitumor Activity as Methuosis Inducers in PC-3 Cells

**DOI:** 10.3390/md22050218

**Published:** 2024-05-14

**Authors:** Bin Yang, Surun Shao, Mingyi Nie, Qingqing Tie, Xiaoyan Pang, Xiuping Lin, Xuefeng Zhou, Yonghong Liu, Xueni Wang, Yunqiu Li

**Affiliations:** 1 CAS Key Laboratory of Tropical Marine Bio-Resources and Ecology, Guangdong Key Laboratory of Marine Materia Medica, South China Sea Institute of Oceanology, Chinese Academy of Sciences, Guangzhou 510301, China; yangbin@scsio.ac.cn (B.Y.); xypang@scsio.ac.cn (X.P.); xiupinglin@scsio.ac.cn (X.L.); xfzhou@scsio.ac.cn (X.Z.); 2 Pharmacy School, Guilin Medical University, Guilin 541004, China; shaosurun@163.com (S.S.); 13438454556@163.com (Q.T.); 3 Guangxi Zhuang Yao Medicine Center of Engineering and Technology, Guangxi University of Chinese Medicine, Nanning 530200, China

**Keywords:** marine fungus, *Peniophora* sp., cytochalasins, citrinin, peniotrinin, anti-prostate methuosis inducer

## Abstract

Two new cytochalasin derivatives, peniotrinins A (**1**) and B (**2**), three new citrinin derivatives, peniotrinins C–E (**4**, **5**, **7**), and one new tetramic acid derivative, peniotrinin F (**12**), along with nine structurally related known compounds, were isolated from the solid culture of *Peniophora* sp. SCSIO41203. Their structures, including the absolute configurations of their stereogenic carbons, were fully elucidated based on spectroscopic analysis, quantum chemical calculations, and the calculated ECD. Interestingly, **1** is the first example of a rare 6/5/5/5/6/13 hexacyclic cytochalasin. We screened the above compounds for their anti-prostate cancer activity and found that compound **3** had a significant anti-prostate cancer cell proliferation effect, while compounds **1** and **2** showed weak activity at 10 μM. We then confirmed that compound **3** exerts its anti-prostate cancer effect by inducing methuosis through transmission electron microscopy and cellular immunostaining, which suggested that compound **3** might be first reported as a potential anti-prostate methuosis inducer.

## 1. Introduction

Fungi are generally considered as a prominent source of bioactive compounds. Generally, fungi, such as *Aspergillus* and *Penicillium* sp., have been used as prolific resources of pharmacologically important secondary metabolites. In contrast, uncommon fungi are underestimated regarding their biosynthetic capacities. As one of the oldest genera of corticoid fungi, the genus *Peniophora* adopted a wide distribution from boreal to tropical areas, and many unrelated species were described in the genus [1]. However, little is known about the production and the pharmaceutical impact of their secondary metabolites. Some numbered examples have been obtained from several *Peniophora* species, such as incarnatin A from *Peniophora incarnate* [2], incarxanthones A–F from *Peniophora incarnata* Z4 [3], and five new drimane sesquiterpenes from *Peniophora polygonia* [4].

Prostate cancer (PCa) is the most commonly diagnosed malignancy and was the second-leading cause of cancer mortalities in the United States in 2023 [5]. Over the past few decades, the treatment of PCa improved greatly, which has effectively improved the overall survival of patients. However, there is still an urgent need for effective new drugs for drug-resistant PCa. Structurally diverse anti-cancer drugs have been developed based on marine natural products or their derivatives. During our search for novel and bioactive compounds from marine sources, several compounds have been reported as potential anti-prostate candidates with remarkable pharmaceutical potential, including dankasterone A [6] and 5′-epiequisetin [7]. The methuosis, which is characterized by vacuole accumulation in the cytoplasm, is a new type of nonapoptotic cell death. Although the precise molecular mechanism is not clear, few methuosis inducers have been reported, such as MOMIPP and Vacquinol-1 in glioblastoma [8,9], as novel therapeutics for the treatment of cancer. However, no methuosis inducer in PCa has been reported.

**Figure 1:**
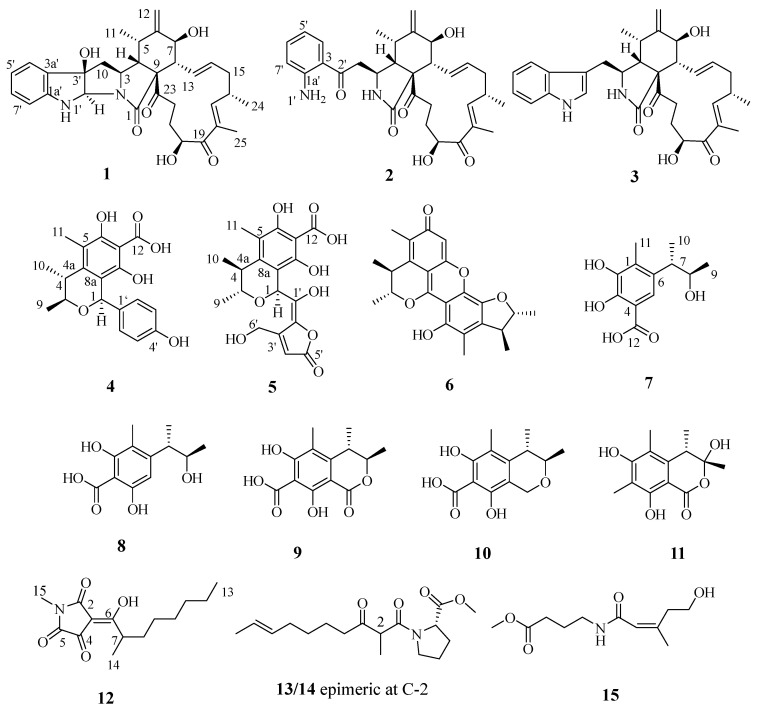
Chemical structures of compounds **1**–**15**.

In our search for secondary metabolites from marine-derived fungi, a *Peniophora* sp. strain SCSIO41203, isolated from an unidentified soft coral sample collected from the Weizhou Island (Guangxi, China), Beibu Gulf of the South China Sea, was selected for its interesting HPLC-UV profile. In the chemical study of this strain, three cytochalasin derivatives, eight citrinin derivatives, one tetramic acid derivative, and three other metabolites were isolated (Figure 1). Among them, **1**, **2**, **4**, **5**, **7,** and **12** were new compounds. Herein, we report the isolation, structural elucidation, and bioassay screening of these compounds.

## 2. Results

**Figure 2:**
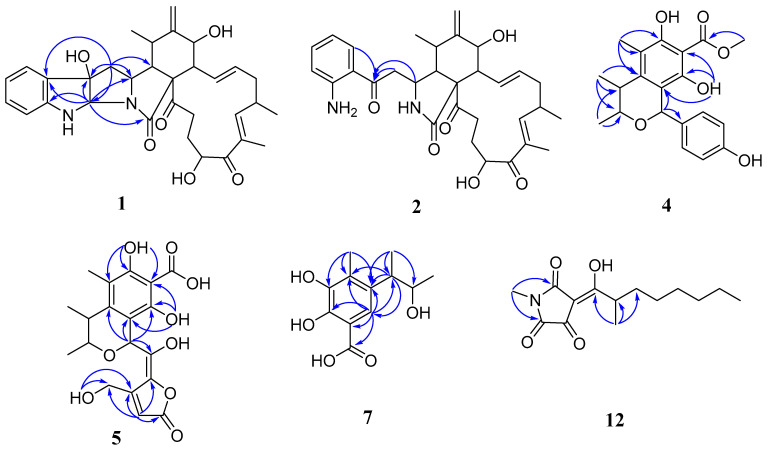
Selected HMBC correlations of compounds **1**, **2**, **4**, **5**, **7** and **12**.

**Figure 3:**
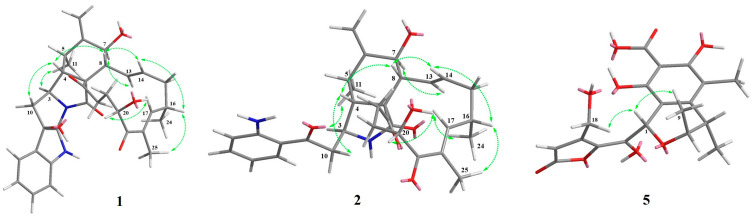
Key NOESY correlations of compounds **1**, **2**, and **5**.

**Figure 4:**
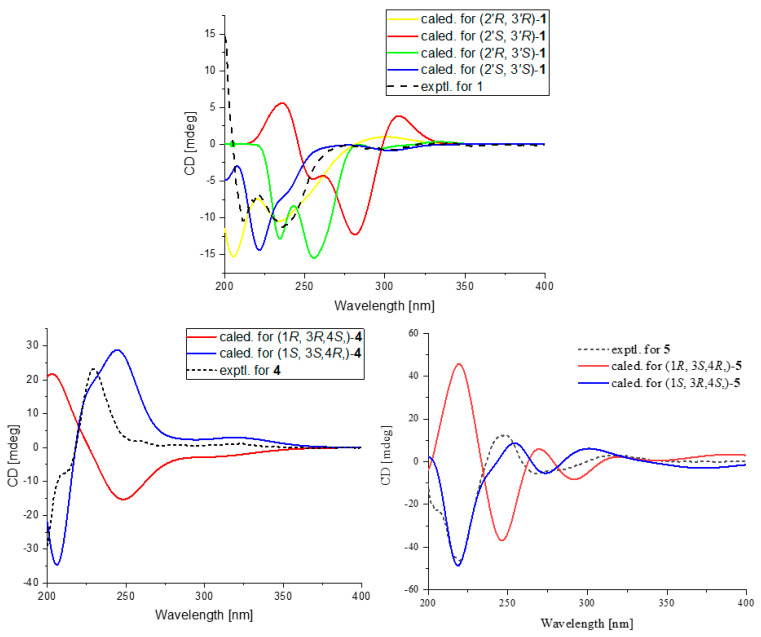
Experimental and calculated ECD spectra compounds **1**, **4**, and **5**.

Compound **1** was obtained as a pale brown oil. The molecular formula of **1** was established as C_32_H_38_N_2_O_6_, based on the molecular ion peaks at *m*/*s* 547.2806 in the HRESIMS spectrum, requiring 15 degrees of unsaturation. The ^13^C NMR spectrum, combined with the HSQC spectrum, ascribed 32 carbon resonance signals to three methyls, five methylenes, fifteen methines including seven olefinic carbons, three oxygenated methines, and nine non-protonated carbons including four olefinic carbons, one oxygen-bearing quaternary carbon, two ketones at *δ*_C_ 204.3, 206.9 and one carbonyl at *δ*_C_ 171.8. The NMR spectral data of **1** (Table 1) reveal a close similarity to those reported for chaetoglobosin Fex (**3**) [10,11]. However, the close comparison of the ^13^C NMR spectroscopic data of **1** and the known compound revealed some differences: one trisubstituted double bond in the latter was changed to another five-membered ring, fused to the isoindole ring at N-2. This assumption was supported by the correlation of H-10 to C-2′, C-3′, and C-3a′, H-2′ to C-1a′, C-3′, C-3a′, C-1, and C-3 in the HMBC spectrum (Figure 2), together with the HRESIMS data. However, **1** represents a new type of cytochalasin bearing a rare 6/5/5/5/6/13 hexacyclic skeleton (Figure 1). The relative configuration of **1** was deduced according to proton coupling constants (Table 1) and the NOESY experiment (Figure 3). The NOESY correlations among H-10, H-4, H-5, and H-8 suggested that their *β* orientations and the boat conformation of the cyclohexane ring shared the same configurations with chaetoglobosin Fex [12]. In addition, the NOESY correlations of H-15*α*/H-13, H-17, and H-17/H-20 indicated those protons in closeness in space, which suggested H-20 was *β*-oriented. The configuration of H-16 was confirmed by the NOESY correlation of H-16/H_3_-25. The coupling constants (*J*_13,14_ = 13.65 Hz) indicated the *E* configuration of the C-13 alkene. Moreover, the correlations from H-16 to Me-25, and H-17 to H-15*α*in the NOESY spectrum led to the assignment of the sharing *E* configuration of the ∆^17,18^ double bond [13]. The stereochemistry of the C-2′ and C-3′ was designated as 2′*R* and 3′*R* by comparing with the experimental ECD curve (Figure 4) after unsuccessful attempts to obtain single crystals of **1**. Based on the above data, **1** was deduced as a rare 6/5/5/5/6/13 hexacyclic cytochalasin, which was named peniotrinin A.

**Scheme 1:**
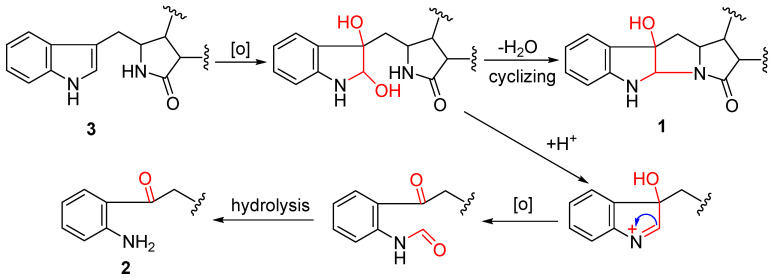
Plausible Biogenetic Pathway of compounds **1**–**3**.

Compound **2** was isolated as pale brown oil with the molecular formula of C_31_H_38_N_2_O_6_ as determined by HRESIMS peak at *m*/*z* 535.2805 [M + H]^+^ (calcd for C_31_H_39_N_2_O_6_, 535.2803), implying 14 degrees of unsaturation. The 1D-NMR data (Table 1) aided with HSQC spectrum of **2** revealed the typical characteristic of a chaetoglobosin scaffold with three methyls, five methylenes, fourteen methines, and nine non-protonated carbons (including four olefinic carbons, three ketones, and one carbonyl). The above NMR data highly resembled those of **1**. The detailed NMR analysis revealed that the carbon at the 2′-position of the indole unit in **1** was missing in **2**, and the oxygen-bearing quaternary carbon at the 3′-position in **1** was replaced by a ketone carbon at *δ*_C_ 199.7 in **2**, which was also verified by the HMBC correlation (Figure 2) from H-4′, H-3, and H-10 to C-2′. The relative configurations of the chiral centers of **2** were established to be the same as those of **1** based on their NOESY spectra (Figure 3). The same relative configuration, similar experimental ECD curve, and similar specific options among **1, 2,** and **3** indicated they also shared an identical absolute configuration. Compound **2** also contains a rare degraded indole skeleton similar to that present in oxi-chaetoglobosin I, which is derived from chaetoglobosin W with oxi-chaetoglobosins G and H both serving as its biosynthetic intermediates [14]. A similar hypothetic biogenetic pathway of **1**–**3** is also proposed (Scheme 1). However, compound **1** represents a novel type of cytochalasin bearing a unique 5-membered carbocyclic ring, which is significantly different from the C-2′/C-3′-epoxide of oxi-chaetoglobosins G and H.

Compound **4** was isolated as a brownish solid. Its molecular formula was determined as C_19_H_20_O_6_ by the positive HRESIMS (*m*/*z* 345.1334 [M + H]^+^, calcd for 345.1333), indicating 10 degrees of unsaturation. The 1D-NMR data (Table 2) aided with HSQC spectrum of **4** showed resonances for three exchangeable protons, three methyl groups, seven methines (including four olefinic carbons, and two oxymethines), and nine non-protonated carbons (including eight olefinic carbons, and one carbonyl). Analysis of the ^1^H and ^13^C NMR spectra of **4** (Table 2) showed the characteristic chemical signals for a 1, benzene group attached to C-1 of the dihydrocitrinin moiety. This assumption was supported by the correlation of CH_3_-9 to C-3, and C-4, CH_3_-10 to C-3, C-4, and C-4a, CH_3_-11 to C-4a, C-5, and C-6, OH-8 to C-7, C-8, and C-8a, H-1 to C-8a, and C-1′ in the HMBC spectrum (Figure 2). Hence, the planar structure of **4** was determined, as shown in Figure 1. In the NOESY spectrum, there was no NOE correlation between H-1 and CH_3_-9 (Figure 3). The absolute configuration of **4** was designated as 1*S*, 3*S*, and 4*R* by comparing with the experimental ECD curve (Figure 4) after unsuccessful attempts to obtain single crystals of **4**. Taken together, the structure of **4** was determined and given the trivial name peniotrinin C.

Compound **5** was determined to have a molecular formula of C_19_H_20_O_9_ based on HRESIMS data at *m*/*z* 393.1178 [M + H]^+^, accounting for ten degrees of unsaturation. The ^1^H and ^13^C NMR data (Table 2) suggested that **5** had a dihydrocitrinin skeleton. The ^13^C NMR spectra also revealed the presence of other 6 carbon signals including a hydroxymethylene at *δ*_H_ 4.12 (dq, *J* = 8.4 Hz, H-6′), and *δ*_C_ 59.9 (C-6′), three olefinic quaternary carbons at *δ*_C_ 143.2 (C-2′), 150.1 (C-1′), and 167.5 (C-3′), one olefinic methine at *δ*_H_ 6.26 (s, H-4′), and *δ*_C_ 109.0, and an extra carbonyl carbon at *δ*_C_ 174.7. HMBC correlations from H-4′ to C-2′, C-3′, C-5′, and C-6′, and H-6′ to C-2′, C-3′, and C-4′ confirmed the substructure. The connection of this substructure with C-1 was confirmed by the HMBC correlation of H-1 to C-1′ (Figure 2). The above functionalities account for nine of the ten degrees of unsaturation in the molecule, revealing another cyclic structure for **5**. In the ^1^H NMR spectrum of **5**, recorded in DMSO-*d*_6_, four additional hydroxyls at *δ*_H_ 14.66, 14.60, 8.73, and 5.55 were observed. The HMBC correlations from 6-OH to C-5, C-6 and C-7, 8-OH to C-7, C-8 and C-9, 6′-OH to C-3′, and C-6′, confirmed that these positions contained free hydroxyl groups. Considering the molecular formula of **5,** the chemical shift for C-1′ and C-2′ dictated that they could be oxygenated, thus either the C-1′ or the C-2′ oxygen must be linked to carbonyl carbon C-5′ to form a lactone ring. Given the partial structures and the observations listed above, the two alternate structures are shown in Figure S47 At this point, the available evidence was incapable of unambiguously determining which of these two structures was correct, and the free hydroxyl proton did not show an HMBC correlation with any of the carbons. The NMR calculations of two candidate structures (5a and 5b) were then carried out using the gauge independent atomic orbital (GIAO) strategy at the B3LYP/6-31+G (d,p) level of theory in the PCM solvent continuum model. DP4^+^ analysis of calculated ^1^H & ^13^C NMR data of **5a** and **5b** were in accordance, and clearly noted 5b as the most probable stereoisomer in high confidence (99.98%) (Tables S1–S3). The configuration of the ∆^13,14^ double bond was established as *Z* through NOESY correlations observed between the H-6′ and H-1 (Figure 2). The NOE correlations of H-1 to CH_3_-9 were observed in its NOESY spectrum (Figure 3). The absolute configurations of compound **5** were determined by a comparison of the experimental ECD data with the calculated ECD data, indicating the absolute configuration of **5** to be 1*S*, 3*R*, and 4*S* (Figure 4). Thus, the chemical structure of compound **5** was assigned as peniotrinin D.

Compound **7** was obtained as a pale yellow oil, and the molecular formula was determined to be C_12_H_16_O_5_, as evidenced by a sodium adduct ion at *m*/*z* 239.0927 in its HRESIMS spectrum, indicating four degrees of unsaturation. The ^1^H NMR spectrum of **8** revealed characteristic signals for an aromatic singlet at *δ*_H_ 6.50 (s, H-5), two methines at *δ*_H_ 3.87 (t, *J* = 6.0 Hz, H-8), 3.09 (t, *J* = 6.5 Hz, H-7), of which one is oxygenated, and three methyls at *δ*_H_ 2.17 (s, H-11), 1.19 (d, *J* = 6.5 Hz, H-10), and 1.14 (d, *J* = 6.0 Hz, H-9). The ^1^H and ^13^C NMR data of **7** revealed its structural similarity to phenol A acid (**8**) [15,16,17]. Detailed analysis of the 1D NMR data for compounds **7** and **8** revealed that differences in the chemical shifts of C-3, C-4, C-5, C-6, and C-12 were observed. In the HMBC spectrum (Figure 2), the correlations from H-5 to C-12 suggested the difference in the position of the carbonyl group, which was attached to C-4. The similar experimental ECD curve and similar specific options between **7** and **8** indicated they also shared an identical absolute configuration. Finally, compound **7** was named peniotrinin E.

Compound **12** was obtained as a pale brown oil. The molecular formula of **12** was established as C_14_H_21_NO_4_, based on the molecular ion peaks at *m*/*z* 268.1541 [M + H]^+^ in the HRESIMS spectrum, requiring five degrees of unsaturation. Analysis of the ^13^C NMR data with the aid of the HSQC spectra resolved all 14 carbon resonances in the molecule, including three methyls, five methylenes, one methine, and five non-protonated carbons. The NMR data of **12** showed a 3-acyl tetramic acid ring, a 2-isooctyl moiety, and an N-methyl group, which was similar to those of penicillenol A_1_ [18,19], except for the presence of one carbonyl carbon at *δ*_C_ 171.3 in **12** and the disappearance of one oxygenated methane and one methyl. This assumption was confirmed by HMBC correlations from CH_3_-15 to C-2 and C-5 and the molecular formula of **12**. However, the stereo configuration of the CH_3_-14 could not be figured out currently. Finally, compound **12** was named peniotrinin F.

Additionally, the structures of these known compounds were mainly elucidated by spectral data analysis as well as comparison with those reported in the literature. They were identified as chaetoglobosin Fex (**3**) [10,11], dicitrinin A (**6**) [15], phenol A acid (**8**) [15], dihydrocitrinone (**9**) [15,20], dihydrocitrinin (**10**) [15], (3*S*, 4*S*) sclerotinin A (**11**) [21,22], tumonoic acids K (**13**) and L (**14**) [23], and ‘alteamide’ (**15**) [24].

**Figure 5:**
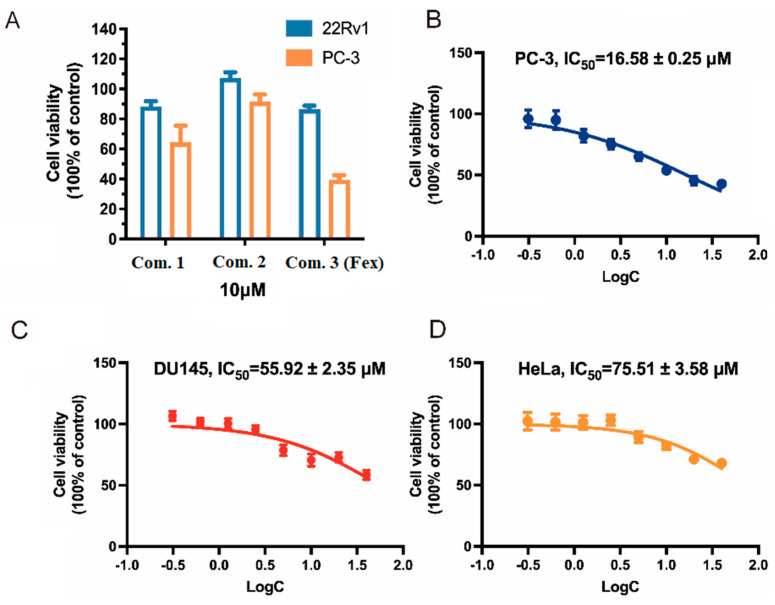
Compound **3** inhibited the cell viability of cancer cells. (**A**) Screening of compounds for their activity against PCa. (**B**) Compound **3** inhibited the cell viability of PC-3 cells. (**C**) Compound **3** inhibited the cell viability of DU145 cells. (**D**) Compound **3** inhibited the cell viability of HeLa cells.

**Figure 6:**
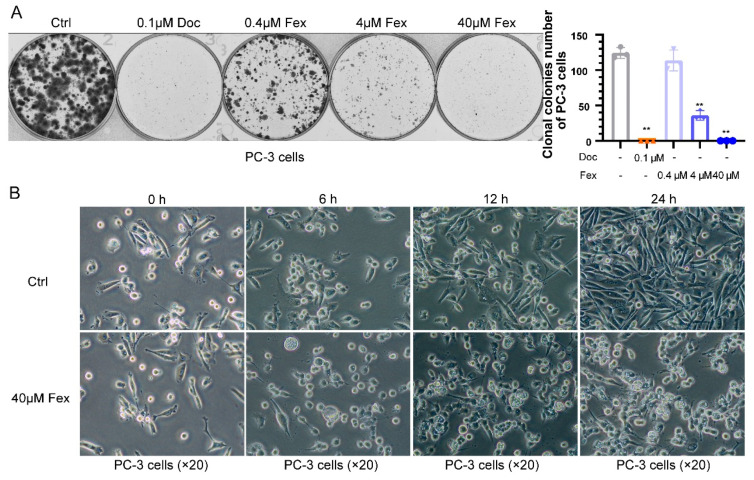
Compound **3** inhibited the proliferation of PC-3 PCa cells. (**A**) Compound **3** inhibited the formation of clonal colonies from PC-3 cells. (**B**) Compound **3** altered the morphology of PC-3 cells. ** *p* < 0.01 vs. control.

We performed a preliminary screening evaluation of the anti-PCa activity of these compounds using two cell models. One was 22Rv1 cells, which are PCa cells that express the androgen receptor, and the other was PC-3 cells, which do not express the androgen receptor. The results of the screening showed that compound **3** had significant anti-PCa activity, and its proliferation inhibition activity against PC-3 cells was significantly better than that of 22Rv1 cells, while compounds **1** and **2** showed weak activity at 10 μM (Figure 5A). We then found that compound **3** inhibited PC-3 cell viability with an IC_50_ of 16.58 ± 0.25 μM by MTT assay (Figure 5B). At the same time, we also tested that compound **3** inhibited cell viability of another PCa cell (DU145), which does not express an androgen receptor, with an IC_50_ of 55.92 ± 2.35 μM (Figure 5C). In addition, we examined the effect of compound **3** on the cell viability of HeLa, a cervical cancer cell, with an IC_50_ of 75.51 ± 3.58 μM (Figure 5D). These data suggest that compound 3 inhibits PCa cell viability to a greater extent than cervical cancer cells. Furthermore, of the three PCa cell lines tested, compound 3 showed the most potent inhibitory activity against PC-3 cells. We then confirmed that compound **3** could inhibit PC-3 proliferation in a dose-dependent manner using a plate clone formation assay (Figure 6A). To understand why compound **3** inhibited PC-3 cell proliferation, we first observed the morphological changes in the cells after compound **3** treatment of PC-3 cells. We observed that after 6 hours of compound **3** treatment of PC-3 cells, more than half of the cells became rounded instead of gradually spreading on the bottom surface of the culture plate-like untreated cells; after 12 h of Compound **3** treatment, the number of rounded cells in the treated group became more, and many vacuoles could be observed in the cells of the treated group; After 24 h of compound **3** treatment, almost all cells in the treated group became rounded and swollen with many vacuoles inside the cells, and the density of the cells was significantly lower than that of the untreated group (Figure 6B). This phenotype suggests that compound **3** may induce the production of vacuoles in the cells, leading to the inability of the cells to survive normally and thus the inability of the cells to proliferate.

**Figure 7:**
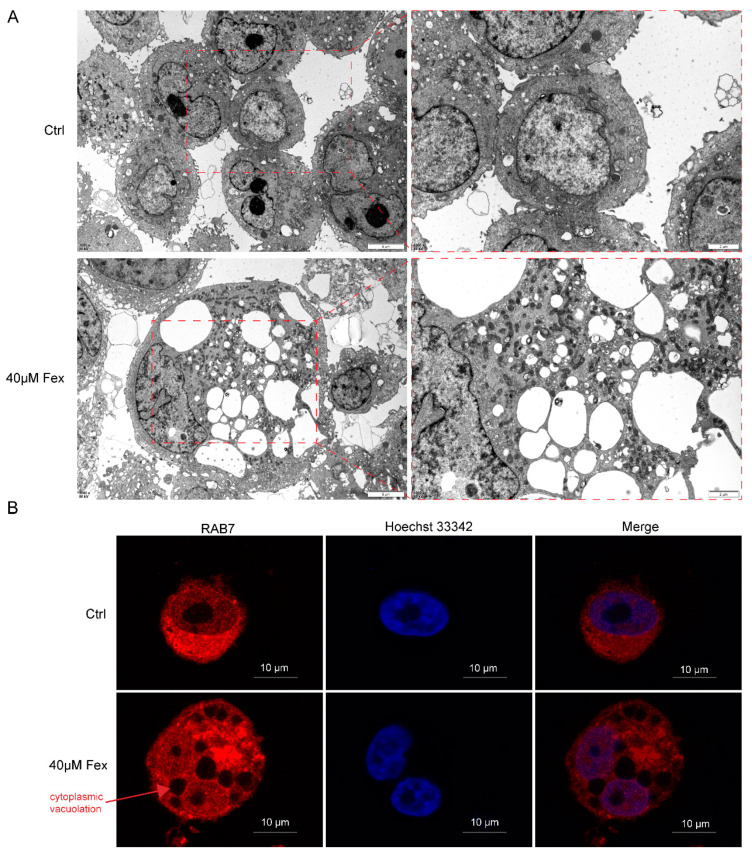
Compound **3** induces methuosis in PC-3 cells. (**A**) Morphology of PC-3 cells under the transmission electron microscope after treatment with compound 3 for 12 h; (**B**) compound **3** produced a large number of vacuoles in PC-3 cells after 12 h of treatment.

To further understand the phenomenon of compound **3** inducing cells to produce vacuoles. We used transmission electron microscopy to observe the changes that occurred in the cells after compound **3** treatment of PC-3 cells for 12 hours. We found that after compound **3** treatment, firstly, the cells were much enlarged, roughly twice the size of the cells in the untreated group; and then there were many huge vacuoles produced inside the cells (Figure 7A). In order to confirm these intracellular vacuoles, we stained the intracellular Rab7 protein by immunofluorescence staining, and the parts without staining color indicated intracellular vacuoles. By this method, we confirmed that a large number of intracellular macrovesicles were indeed produced after compound **3** treatment of the cells (Figure 7B). Combined with previous studies describing methuosis, the cells were enlarged in size and a large number of intracellular vacuoles were produced [8]. We conclude that compound **3** induced methuosis in PC-3 cells, which led to the inability of the cells to proliferate and ultimately exerted anti-PCa effects.

## 3. Materials and Methods

### 3.1. General Experimental Procedures

The NMR spectra were recorded on a Bruker AC 500 or AVANCE III HD 700 NMR spectrometer. Chemical shift values were expressed in *δ* (ppm) downfield from TMS, as an internal standard. The mass spectra, including high-resolution mass spectra, were measured on a Bruker micrOTOF-QII mass spectrometer. Optical rotation values were measured with a PerkinElmer MPC 500 polarimeter. UV spectra were recorded on a Shimadzu UV-2401 PC spectrophotometer (Shimadzu Corporation, Kyoto, Japan). CD spectra were obtained with a Chirascan circular dichroism spectrometer (Applied Photophysics). IR spectra were obtained on Tensor 27 (Bruker Optics GmbH, Ettlingen, Germany) with KBr pellets. Column chromatography (CC) was performed on plates precoated with over silica gel (200–300 mesh) (Qingdao Marine Chemical Factory) and YMC gel (ODS-A, 12 nm, S-50 µm). Semipreparative HPLC was performed using an ODS column (YMC-pack ODS-A, YMC Co., Ltd., 10 × 250 mm, 5 µm, Kyoto, Japan). The silica gel GF254 used for TLC was supplied by the Qingdao Marine Chemical Factory, Qingdao, China. Spots were detected on TLC under UV light or by heating after spraying with 5% H_2_SO_4_ in EtOH (*v*/*v*). Artificial sea salt was a commercial product (Guangzhou Haili Aquarium Technology Company, Guangzhou, China).

### 3.2. Fungal Material

The culture of *Peniophora* sp. SCSIO41203 was isolated from a soft coral sample near Weizhou Island (Guangxi, Beihai, China), Beibu Gulf of the South China Sea. The strain was identified as *Peniophora* sp. based on a molecular biological protocol calling for DNA amplification and ITS region sequence comparison with the GenBank database and shared a similarity of 98% with *Peniophora* sp. MUCC804 (accession NO. KF541333.1). The strain was deposited in the RNAM Center, South China Sea Institute of Oceanology, Chinese Academy of Sciences.

### 3.3. Fermentation and Extraction

Strain stored on PDA slants at 4 °C was cultured on PDA agar plates and incubated for 7 days at 28 °C in the incubator. Seed medium (infusion from 15 g of malt extract powder, sea salt, 2.5 g, distilled water, 1000 mL, pH = 7.4~7.8) in 150 mL Erlenmeyer flasks was inoculated with strain SCSIO41203 and incubated at 25 °C for 3 days on a rotating shaker (180 rpm). Autoclaved rice solid-substrate medium in 1000 mL flasks (rice 200 g, sea salt 6.6 g, water 220 mL) was inoculated with 15 mL seed solution. Flasks were incubated at 28 °C in a static position. After 60 days, cultures from 40 flasks were harvested for the isolation of substances. The obtained solid culture was crushed and extracted with twice the amount of acetone three times. The acetone extract was evaporated under reduced pressure to afford an aqueous solution, and then the aqueous solution was extracted with twice the amount of EtOAc to yield a crude gum (95 g).

### 3.4. Isolation and Purification

The EtOAc crude extract was chromatographed over a silica gel column eluted with PE-EtOAc-MeOH (50:1:0 to 0:0:1, *v*/*v*) in a gradient to yield seventeen fractions (Frs.1~17). Fr.5 was subjected to MPLC with an ODS column and eluted with MeOH/H_2_O (5–100%) to give seven subfractions (Frs.5–1~5–7). Fr.5–3 was separated by semipreparative HPLC (Shimedzu, Kyoto, Japan) (42% MeOH/H_2_O, 2.5 mL/min) to yield **11** (9.1 mg, *t*_R_ = 17.0 min). Fr.5–6 was separated by semipreparative HPLC (30% MeOH/H_2_O, 3 mL/min) to yield **5** (13.4 mg, *t*_R_ = 37.0 min), **8** (6.6 mg, *t*_R_ = 14.5 min), **9** (20.7 mg, *t*_R_ = 26.0 min) and **10** (10.5 mg, *t*_R_ = 51.0 min). Fr.6 was subjected to MPLC with an ODS column and eluted with MeOH/H_2_O (5–100%) to give five subfractions (Frs.6–1~6–5). Fr.6–1 was separated by semipreparative HPLC (34% MeOH/H_2_O, 3 mL/min) to yield **12** (13.9 mg, *t*_R_ = 33.0 min). Fr.6–2 was separated by semipreparative HPLC (35% MeOH/H_2_O, 3 mL/min) to yield **7** (3.9 mg, *t*_R_ = 33.0 min) and **15** (3.1 mg, *t*_R_ = 16.6 min). Fr.6–3 was separated by semipreparative HPLC (50% MeOH/H_2_O, 3 mL/min) to yield **1** (3.2 mg, *t*_R_ = 18.2 min), **2** (3.5 mg, *t*_R_ = 15.2 min) and **3** (8.4 mg, *t*_R_ = 20.1 min). Fr.7 was subjected to MPLC with an ODS column and eluted with MeOH/H_2_O (20–100%) to give three subfractions (Frs.7–1~7–3). Fr.7–2 was separated by semipreparative HPLC (55% MeOH/H_2_O, 3 mL/min) to yield four subfractions (Frs.7–2–1~7–2–4). Fr.7–2–3 was further separated by semipreparative HPLC (35% CH_3_CN/H_2_O, 3 mL/min) to yield **13** (7.3 mg, *t*_R_ = 29.5 min) and **14** (10.4 mg, *t*_R_ = 32.5 min). Fr.9 was subjected to MPLC with an ODS column and eluted with MeOH/H_2_O (20–100%) to give five subfractions (Frs.9–1~9–5). Fr.9–5 was separated by semipreparative HPLC (30% MeOH/H_2_O, 3 mL/min) to yield **4** (4.1 mg, *t*_R_ = 21.3 min). Fr.11 was subjected to MPLC with an ODS column and eluted with MeOH/H_2_O (20–100%) to give seven subfractions (Frs.11–1~11–7). Fr.11–2 was separated by semipreparative HPLC (55% MeOH/H_2_O, 3 mL/min) to yield **6** (21.0 mg, *t*_R_ = 38.5 min).

Peniotrinin A (**1**): Pale brown oil; [*α*] $\overset{25}{D}$ = −117.4 (*c* = 0.22, MeOH); CD (MeOH; *c* 0.2): *λ*_max_ (Δ*ε*) 236 (−9.34), 220 (−5.59), 211 (−8.58); UV (MeOH) *λ*_max_ (log *ε*): 205.2 (1.355) nm; IR *ν*_max_ (film) 3352, 2916, 2850, 1668, 1404, 1089, 1018, 950, 744, 599 cm^−1^; ^1^H and ^13^C NMR data, Table 1; HRESIMS *m*/*z* 547.2806 [M + H]^+^ (calcd for C_32_H_39_N_2_O_6_, 547.2803), 569.2658 [M + Na]^+^ (calcd for C_32_H_38_N_2_NaO_6_, 569.2622).

Peniotrinin B (**2**): Pale brown oil; [*α*] $\overset{25}{D}$ = −50.1 (*c* = 0.25, MeOH); CD (MeOH; *c* 0.2): *λ*_max_ (Δ*ε*) 368 (−2.33), 280 (+0.19), 222 (−8.04); UV (MeOH) *λ*_max_ (log *ε*): 367.4 (0.222), 226.6 (1.413), 203.8 (1.419) nm; IR *ν*_max_ (film) 3334, 2918, 2833, 1681, 1653, 1456, 1018, 599 cm^−1^; ^1^H and ^13^C NMR data, Table 1; HRESIMS *m*/*z* 535.2805 [M + H]^+^ (calcd for C_31_H_39_N_2_O_6_, 535.2803), 557.2633 [M + Na]^+^ (calcd for C_31_H_38_N_2_NaO_6_, 557.2622).

Peniotrinin C (**4**): Brownish solid; [*α*] $\overset{25}{D}$ = +150.8 (*c* = 0.20, MeOH); CD (MeOH; *c* 0.2): *λ*_max_ (Δ*ε*) 230 (+12.12), 209 (−4.05); UV (MeOH) *λ*_max_ (log *ε*): 319.2 (0.352), 253.0 (0.753), 214.0 (2.421) nm; IR *ν*_max_ (film) 3365, 2968, 2929, 1585, 1512, 1402, 1354, 1257, 1024, 835, 761, 682 cm^−1^; ^1^H and ^13^C NMR data, Table 2; HRESIMS *m*/*z* 345.1334 [M + H]^+^ (calcd for C_19_H_21_O_6_, 345.1333), 367.1169 [M + Na]^+^ (calcd for C_19_H_20_NaO_6_, 367.1152).

Peniotrinin D (**5**): Brownish solid; [*α*] $\overset{25}{D}$ = −308.3 (*c* = 0.50, MeOH); CD (MeOH; *c* 0.2): *λ*_max_ (Δ*ε*) 316 (+1.83), 267 (−3.24), 248 (+7.43), 220 (−27.54); UV (MeOH) *λ*_max_ (log *ε*): 253.6 (0.651), 213.2 (1.720) nm; IR *ν*_max_ (film) 3344, 2949, 2835, 2358, 1653, 1647, 1014, 667, 553 cm^−1^; ^1^H and ^13^C NMR data, Table 2; HRESIMS *m*/*z* 393.1178 [M + H]^+^ (calcd for C_19_H_21_O_9_, 393.1180), 415.0997 [M + Na]^+^ (calcd for C_19_H_20_NaO_9_, 415.1000).

Peniotrinin E (**7**): Pale brown oil; [*α*] $\overset{25}{D}$ = −43.0 (*c* = 0.10, MeOH); *λ*_max_ (Δ*ε*) 262 (−0.50), 242 (−0.25), 210 (−2.61); UV (MeOH) *λ*_max_ (log *ε*): 322.6 (0.757), 256.8 (2.656), 222.2 (3.658); IR *ν*_max_ (film) 2972, 2358, 1633, 1415, 1012, 995, 759 cm^−1^; ^1^H NMR (500 MHz, DMSO-*d*_6_) *δ*: 6.50 (1H, s, H-5), 3.87 (1H, t, *J* = 6.0 Hz, H-8), 3.09 (1H, t, *J* = 6.5 Hz, H-7), 2.17 (3H, s, H-11), 1.19 (3H, d, *J* = 6.5 Hz, H-10), 1.14 (3H, d, *J* = 6.0 Hz, H-9); ^13^C NMR (125 MHz, DMSO-*d*_6_) *δ*: 172.8 (C-12), 159.1 (C-2), 157.3 (C-3), 152.3 (C-6), 114.3 (C-1), 105.4 (C-5), 98.5 (C-4), 69.6 (C-8), 42.3 (C-7), 20.5 (C-10), 16.4 (C-9), 10.6 (C-11); HRESIMS *m*/*z* 239.0927 [M − H]^−^ (calcd for C_12_H_15_O_5_, 239.0925), 479.1921 [2M − H]^−^ (calcd for C_24_H_31_O_10_, 479.1923).

Peniotrinin F (**12**): Pale brown oil; [*α*] $\overset{25}{D}$ = −26.7 (*c* = 0.20, MeOH); UV (MeOH) *λ*_max_ (log *ε*): 347.2 (0.223), 258.6 (1.746), 239.6 (1.245), 202.2 (1.105) nm; IR *ν*_max_ (film) 3340, 2941, 2833, 1651, 1456, 1022, 682, 549 cm^-1^; ^1^H NMR (500 MHz, DMSO-*d*_6_) *δ*: 3.60 (1H, m, H-7), 2.91 (3H, s, H-15), 1.60 (1H, m, H-8a), 1.34 (1H, m, H-8b), 1.28 (8H, m, H-9, H-10, H-11, H-12), 1.05 (3H, d, *J* = 7.0 Hz, H-14), 0.87 (3H, t, *J* = 6.5 Hz, H-13); ^13^C NMR (125 MHz, DMSO-*d*_6_) *δ*: 191.9 (C-6), 177.9 (C-4), 171.3 (C-5), 164.1 (C-2), 100.5 (C-3), 38.1 (C-7), 33.6 (C-8), 31.6 (C-12), 29.2 (C-10), 27.2 (C-9), 23.5 (C-15), 22.5 (C-11), 17.5 (C-14), 14.4 (C-13). HRESIMS *m*/*z* 268.1541 [M + H]^+^ (calcd for C_14_H_22_NO_4_, 268.1543), 290.1361 [M + Na]^+^ (calcd for C_14_H_21_NNaO_4_, 290.1363), 557.2826 [2M + Na]^+^ (calcd for C_28_H_42_N_2_NaO_8_, 557.2833).

### 3.5. Computational Methods

The theoretical ECD curve of new compounds was calculated by the Gaussian 09 software. Molecular Merck force field (MMFF) was performed with Spartan’14 software. The calculated ECD spectra were generated the same as reported previously [10].

Conformational analysis of the isomers of **5** was carried out by means of the Spartan 14 software using the Merck Molecular Force Field (MMFF) method. The conformers with Boltzmann-population of over 1% (the relative energy within 7 kcal/mol) were reoptimized using density functional theory (DFT) at the B3LYP/6-31+G (d) level under vacuum using the Gaussian 16 program. The stable conformers of diastereomers of **5** were further chosen for ^1^H and ^13^C NMR chemical shift calculations using the gauge including atomic orbital (GIAO) method at the PCM//mPW1PW91/6-311+g(d,p) level of theory for DP4^+^ calculations. The calculations in solution were carried out using the polarizable continuum model, PCM, for DMSO (the solvent used experimentally). The unscaled chemical shifts (*δ*u) were computed using TMS as the reference standard using the following equation: *δ*u = σ0 − σx, where σx is the Boltzmann averaged shielding tensor and σ0 is the shielding tensor of TMS computed at the same level of theory employed for σx. For methyl groups, averages of the computed values of the three hydrogens were used to compare with the experimental data. The DP4^+^ calculations were carried out using the Excel spreadsheet available for free at sarotti-NMR.weebly.com (accessed on 2023 June 11).

### 3.6. Cell Culture

Human PCa 22Rv1, PC-3, DU145 cells, and human cervical carcinoma HeLa cells were obtained from the Cell Bank/Stem Cell Bank of the Chinese Academy of Sciences (Shanghai, China). 22Rv1 cells were grown in RPMI1640 (Gibco, Suzhou, China) supplemented with 10% (*v*/*v*) fetal bovine serum (FBS) (Sigma-Aldrich, Uruguayan origin, St. Louis, MO, USA), 100 μ/mL penicillin and 100 μg/mL streptomycin. DU145 cells were cultured in MEM plus supplemented with the same supplements. PC-3 and HeLa cells were cultured in DMEM F12 plus supplemented with the same supplements. All cell lines were tested and found to be free of mycoplasma contamination. All cells were incubated at 37 °C in a cell incubator containing 5% CO_2_ and the solution was changed once for 2–3 days. All cell lines were identified by STR, and the cells used in the experiment were within 15 passages.

### 3.7. MTT Assay

The MTT assay was performed as described in the previous study [25]. In brief, the cells in the logarithmic growth phase were seeded in 96 plates at a density of 5 × 10^3^ cells per well. After 24 h of cell attachment, the cells were treated with different concentrations of compounds and 0.1% DMSO, respectively, for 72 h, with six replicates for each treatment group. Then 10 μL of MTT solution (5 mg/mL) was added to each well and the cells were returned to the incubator and incubated for a further 4 h. At the end of the incubation, the entire culture solution was gently aspirated from the cells and 100 μL of DMSO solution was added to each well. The solution in the 96-well plate was then shaken well and mixed, and the absorbance of each well was measured at 570 nm by using a hybrid plate reader (Bio Tek, Winooski, VT, USA). GraphPad Prism 8.0 software was used to fit the IC_50_ for compound **3** inhibition of cell viability.

### 3.8. Plate Clone Formation Experiment

PC-3 cells were seeded in 6-well plates at a density of 1000 cells per well for 48 h. The cells were then treated with DMSO (0.1%, *v*/*v*), docetaxel (0.1 μM) and Compound **3** (0.4, 4 and 40 μM). The culture medium was changed once a week and after two weeks the cells formed clear clonal colonies, then the cells were fixed for staining. First, the cells were fixed with 4% tissue fixative for 30 min, then the 4% tissue fixative was discarded and the cells were washed with PBS buffer and stained with crystal violet staining solution. After the cells had been stained for 30 min, the staining solution was discarded, the cells were washed with PBS buffer and finally the colonies were photographed and analyzed by using a colony counter (GelCount, Oxford Optronix, Abingdon, UK). The experiment was repeated three times independently.

### 3.9. Transmission Electron Microscopy to Observe Cell Morphology

PC-3 cells were seeded into 10 cm cell culture dishes and incubated with 10% FBS DMEM/F12 for 48 h. PC-3 cells were treated with DMSO (0.1%, *v*/*v*), compound **3** (40 μM) respectively for 12 h. PC-3 cells were pre-fixed in 2.5% glutaraldehyde, post-fixed in 1% osmium tetroxide, dehydrated through a graded series of acetone and embedded in Epon812. Semi-thin sections were stained with toluidine blue for optical localization, and then ultrathin sections were made with a diamond knife to prepare ultrathin sections of about 60~90 nm, spread and then fished on copper mesh 200 mesh square waffle copper mesh; stained with uranyl acetate and lead citrate for 15 min, and then finally the sections were observed by transmission electron microscope (JEM-1400FLASH, JEOL, Tokyo, Japan).

### 3.10. Cellular Immunofluorescence Staining

Cellular immunostaining was performed according to the method of our previous study [25]. Briefly, PC-3 cells were cultured on 35 mm confocal dish pre-treated with 10% l-polylysine. After treatment with compound **3** for 12 h. The cells were then subjected to a series of treatments including fixation, permeabilization, washing and containment. The cells were then incubated with Rab7 (A12308, ABclonal, Wuhan, China) antibody overnight at 4 °C, followed by incubation with Cy3 Goat anti-rabbit IgG (H + L) (AS007, ABclonal) for 1 h at room temperature. The nuclei were then stained with Hoechst 33342 for 5 min and the cells were rinsed with PBS. The cells were analyzed using a Laser Scanning Confocal Microscope (TCS SP5, Leica, Wetzlar, Germany) to obtain images.

## 4. Conclusions

In conclusion, sixteen diverse compounds were isolated and identified from the marine fungus *Peniophora* sp. SCSIO41203. Their structures including absolute configurations were determined by spectroscopic analyses, quantum chemical calculations, and ECD calculations. Cytochalasins have continuously aroused considerable attention due to their structural complexities and pharmacological significance [13,26]. This study investigated two new cytochalasin derivatives peniotrinins A (**1**) and B (**2**), which derived from the known compound chaetoglobosin Fex (**3**). We also isolated and identified eight citrinin derivatives (**4**–**11**), including three new derivatives from this fungus. Interestingly, among these compounds, compound **3** produced significant anti-PCa effects by inducing methuosis. In contrast, not many compounds have been reported to induce methuosis, and no marketed anti-tumor drug is based on this mechanism of action. If compounds based on this mechanism can be developed into drugs, they could be used as an alternative treatment after resistance to chemotherapeutic drugs has led to therapeutic failure and, therefore, such compounds are worthy of further investigation in the development of new anti-tumor drugs. Collectively, these studies show that the species *Peniophora* sp. produces an eccentric diversity of secondary metabolites.

## Figures and Tables

**Table 1:** ^1^H (700 MHz) and ^13^C (175 MHz) NMR spectroscopic data of **1** and **2** in CD_3_OD.

No.	1		2	
	*δ*c, Type	*δ*_H_ (*J* in Hz)	*δ*c, Type	*δ*_H_ (*J* in Hz)
1	171.8, C		175.2, C	
3	59.0, CH	3.19, dt (11.2, 4.9)	48.8, CH	3.66, m
4	46.0, CH	2.73, dd (4.2)	48.2, CH	2.62, dd (5.6, 2.8)
5	31.9, CH	2.67, m	31.8, CH	2.87, m
6	150.5, C		149.4, C	
7	68.8, CH	3.89, m	70.6, CH	3.89, d (10.5)
8	48.4, CH	2.82, dd (11.9, 9.8)	48.4, CH	2.73, dd (9.8)
9	67.1, C		63.0, C	
10	48.1, CH_2_	2.57, dd (11.9, 5.6)	45.3, CH_2_	3.08, dd (14.7, 4.9)
		2.03, dd (11.2)		3.03, dd (16.1, 7.7)
11	14.6, CH_3_	1.02, d (7.0)	12.4, CH_3_	1.05, d (5.6)
12	111.4, CH_2_	5.34, s	113.1, CH_2_	5.28, s
		4.99, s		5.11, s
13	128.5, CH	5.85, ddd (13.7, 10.9, 2.1)	128.0, CH	6.21, ddd (14.4, 10.2, 2.1)
14	134.7, CH	5.37, m	134.3, CH	5.34, ddd (13.3, 13.3, 2.8)
15	40.6, CH_2_	2.53, m	40.6, CH_2_	2.50, m
		2.13, dt (13.3, 11.9)		2.09, m
16	33.5, CH	2.89, m	33.3, CH	2.86, m
17	149.1, CH	6.42, dd (9.1, 0.7)	149.1, CH	6.30, dd (9.8, 1.4)
18	135.0, C		135.4, C	
19	204.3, C		204.3, C	
20	70.8, CH	4.97, m	70.6, CH	4.89, m
21	31.0, CH_2_	1.89, m	30.5, CH_2_	1.90, m
		1.81, m		1.87, m
22	36.7, CH_2_	2.90, m	36.1, CH_2_	2.97, ddd (16.1, 9.1, 5.6)
		2.66, m		2.88, m
23	206.9, C		209.2, C	
24	18.7, CH_3_	1.09, d (7.0)	18.7, CH_3_	1.07, d (6.3)
25	10.9, CH_3_	1.85, s	11.0, CH_3_	1.84, d (1.4)
1′a	150.5, C		151.5, C	
2′	78.4, CH	5.28, s	199.7, C	
3′	91.0, C		116.9, C	
3′a	129.2, C			
4′	123.8, CH	7.27, d (7.0)	130.8, CH	7.65, dd (7.7, 1.4)
5′	118.8, CH	6.79, td (7.7, 0.7)	114.8, CH	6.59, td (8.4, 1.4)
6′	129.9, CH	7.15, td (7.7, 0.7)	134.7, CH	7.24, td (8.4, 2.1)
7′	109.5, CH	6.65, d (8.4)	117.0, CH	6.73, dd (8.4, 0.7)

**Table 2:** ^1^H and ^13^C NMR spectroscopic data of **4** and **5** in DMSO-*d*6.

Pos	4 ^a^		5 ^b^	
	*δ*c, Type	*δ*_H_ (*J* in Hz)	*δ*c, Type	*δ*_H_ (*J* in Hz)
1	71.8, CH	5.58, s	64.4, CH	5.88, s
3	71.6, CH	3.53, dt (11.5, 6.5)	73.3, CH	3.93, dt (11.5, 6.3)
4	36.5, CH	2.62, dt (11.5, 7.0)	35.3, CH	2.63, dt (11.5, 7.0)
4a	141.0, C		141.0, C	
5	109.9, C		110.1, C	
6	159.4, C		159.4, C	
7	102.0, C		101.9, C	
8	156.6, C		156.5, C	
8a	111.9, C		107.3, C	
9	20.1, CH_3_	1.14, d (6.5)	18.8, CH_3_	1.13, d (6.3)
10	19.8, CH_3_	1.10, d (6.5)	20.0, CH_3_	1.26, d (7.0)
11	11.1, CH_3_	1.99, s	10.3, CH_3_	1.97, s
12	176.1, C		176.0, C	
1′	133.5, C		150.1, C	
2′	114.8, CH	6.66, d (8.5)	143.2, C	
3′	129.9, CH	6.98, d (8.5)	167.5, C	
4′	156.6, C		109.0, CH	6.26, s
5′	129.9, CH	6.98, d (8.5)	174.7, C	
6′	114.8, CH	6.66, d (8.5)	59.9, CH_2_	4.12, dq (8.4)
6-OH		14.77, s		14.66, s
8-OH		14.65, s		14.60, s
14-OH				8.73, s
18-OH				5.55, t (6.3)

^a^: ^1^H in 500 MHz and ^13^C in 125 MHz; ^b^: ^1^H in 700 MHz and ^13^C in 175 MHz.

## Data Availability

The research data are available in the Supporting Information.

## Supplementary Materials

The following supporting information can be downloaded at https://www.mdpi.com/article/10.3390/md22050218/s1, Figures S1–S46: ^1^H, ^13^C-NMR, HSQC, HMBC, NOESY, HRESIMS, IR, and UV spectra of compounds **1**, **2**, **4**, **5**, **7**, and **12**. Figures S47–S48, and Tables S1–S3: The NMR calculated data of two candidate structures of compound **5**.
