# Supplementary material for: Novel Metabolites from the Marine-Derived Fungus Peniophora sp. SCSIO41203 Show Promising In Vitro Antitumor Activity as Methuosis Inducers in PC-3 Cells
Source: Mar Drugs. 2024 May 14;22(5):218. doi: 10.3390/md22050218 (PMC11123344; doi:10.3390/md22050218)
Supplement: Supplementary file 1 [file marinedrugs-22-00218-s001.zip › marinedrugs-2996198-supplementary.pdf]

Novel metabolites from the marine-derived fungus *Peniophora* sp. SCSIO41203 shows promising in vitro antitumor activity as methuosis inducers in PC-3 cells

Bin Yang [1,†], Surun Shao [2,†], Mingyi Nie [3], Qingqing Tie [2], Xiaoyan Pang [1], Xiuping Lin [1], Xuefeng Zhou [1], Yonghong Liu [1,*], Xueni Wang [3,*] and Yunqiu Li [2,*]

[1] CAS Key Laboratory of Tropical Marine Bio-Resources and Ecology, Guangdong Key Laboratory of Marine Materia Medica, South China Sea Institute of Oceanology, Chinese Academy of Sciences, Guangzhou 510301, China; yangbin@scsio.ac.cn (B.Y.); xypang@scsio.ac.cn (X.P.); xiupinglin@scsio.ac.cn (X.L.); xfzhou@scsio.ac.cn (X.Z.)

[2] Pharmacy School, Guilin Medical University, Guilin 541004, China; shaosurun@163.com (S.S.); 13438454556@163.com (Q.T.)

[3] Guangxi Zhuang Yao Medicine Center of Engineering and Technology, Guangxi University of Chinese Medicine, Nanning 530200, China; niemingyinew@163.com

* Correspondence: yonghongliu@scsio.ac.cn (Y.L.); wangxueni@gxtcmu.edu.cn (X.W.); leeyq88@126.com (Y.L.)

† These authors contributed equally to this work.

Contents

Figure S1. ^{1}H NMR spectrum of **1** (CD_3OD, 700 MHz) 4
Figure S2. ^{13}C NMR spectrum of **1** (CD_3OD, 175 MHz) 4
Figure S3. HSQC spectrum of **1** (CD_3OD) 5
Figure S4. HMBC spectrum of **1** (CD_3OD) 5
Figure S5. NOESY spectrum of **1** (CD_3OD) 6
Figure S6. HRESIMS spectrum of **1** 6
Figure S7. IR spectrum of **1** 7
Figure S8. UV spectrum of **1** 8
Figure S9. ^{1}H NMR spectrum of **2** (CD_3OD, 700 MHz) 9
Figure S10. ^{13}C NMR spectrum of **2** (CD_3OD, 175 MHz) 9
Figure S11. HSQC spectrum of **2** (CD_3OD) 10
Figure S12. HMBC spectrum of **2** (CD_3OD) 10
Figure S13. NOESY spectrum of **2** (CD_3OD) 11
Figure S14. HRESIMS spectrum of **2** 11
Figure S15. IR spectrum of **2** 12
Figure S16. UV spectrum of **2** 13
Figure S17. ^{1}H NMR spectrum of **4** (DMSO-d_6, 500 MHz) 14
Figure S18. ^{13}C NMR spectrum of **4** (DMSO-d_6, 125 MHz) 14
Figure S19. HSQC spectrum of **4** (DMSO-d_6) 15
Figure S20. HMBC spectrum of **4** (DMSO-d_6) 15
Figure S21. NOESY spectrum of **4** (DMSO-d_6) 16
Figure S22. HRESIMS spectrum of **4** 16
Figure S23. IR spectrum of **4** 17
Figure S24. UV spectrum of **4** 18
Figure S25. ^{1}H NMR spectrum of **5** (DMSO-d_6, 700 MHz) 19
Figure S26. ^{13}C NMR spectrum of **5** (DMSO-d_6, 175 MHz) 19
Figure S27. HSQC spectrum of **5** (DMSO-d_6) 20
Figure S28. HMBC spectrum of **5**(DMSO-d_6) 20
Figure S29. NOESY spectrum of **5** (CD_3OD) 21
Figure S30. HRESIMS spectrum of **5** 21

Figure S31. IR spectrum of **5** 22
Figure S32. UV spectrum of **5** 23
Figure S33. ^{1}H NMR spectrum of **7** (DMSO-d_6, 700 MHz)........ 24
Figure S34. ^{13}C NMR spectrum of **7** (DMSO-d_6, 175 MHz) 24
Figure S35. HSQC spectrum of **7** (DMSO-d_6) 25
Figure S36. HMBC spectrum of **7** (DMSO-d_6) 25
Figure S37. HRESIMS spectrum of **7** 26
Figure S38. IR spectrum of **7** 26
Figure S39. UV spectrum of **7** 27
Figure S40. ^{1}H NMR spectrum of **12** (DMSO-d_6, 500 MHz)........ 28
Figure S41. ^{13}C NMR spectrum of **12** (DMSO-d_6, 125 MHz) 28
Figure S42. HSQC spectrum of **12** (DMSO-d_6) 29
Figure S43. HMBC spectrum of **12** (DMSO-d_6) 29
Figure S44. HRESIMS spectrum of **12** 30
Figure S45. IR spectrum of **12** 30
Figure S46. UV spectrum of **12** 31
Figure S47. The NMR calculations of two candidate structures (**5**a and **5**b) 32
Figure S48. The optimized conformers and equilibrium populations of **5**a and **5**b.... 34
Table S1. Energies of **5** at MMFF94 force field 35
Table S2. Energies of **5** at B3LYP/6–31+G(d, p) level in methanol 36
Table S3. DP4+ analysis of calculated ^{1}H & ^{13}C NMR data of **5a** and **5b**
(experimental for **5**, isomers **1** and **2** for **5a** and **5b**, respectively) 37

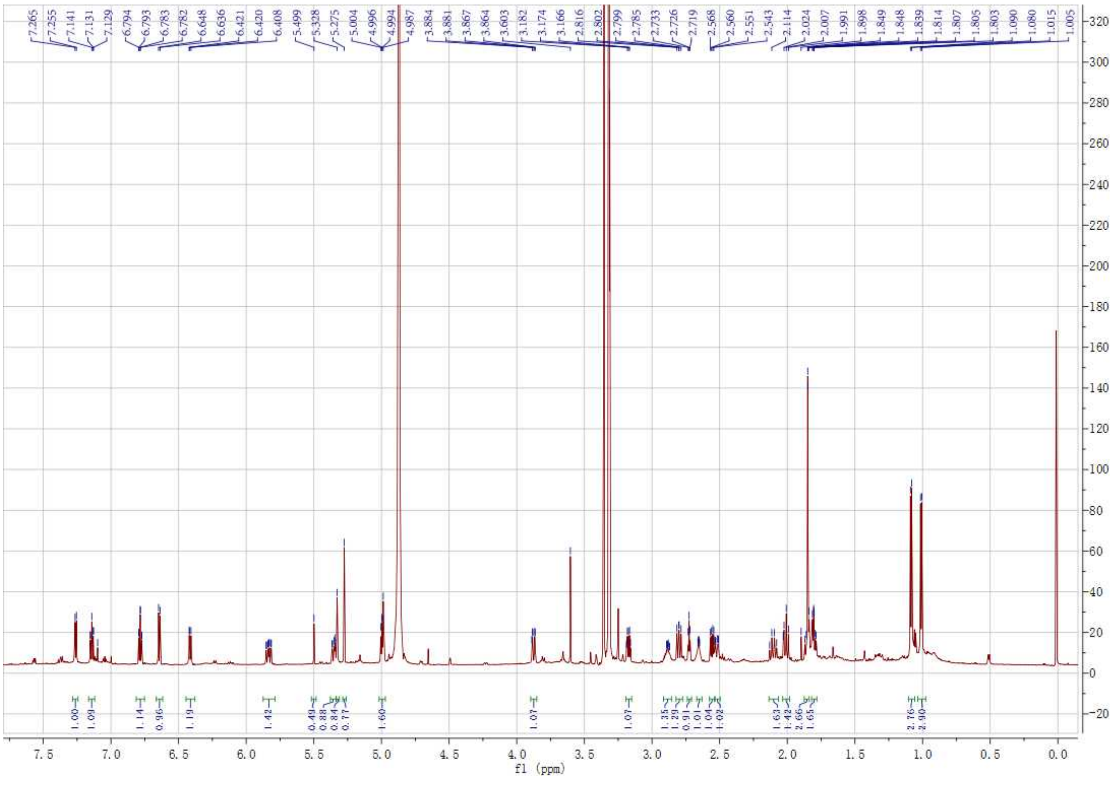

Figure S1. ^{1}H NMR spectrum of **1** (CD_3OD, 700 MHz)

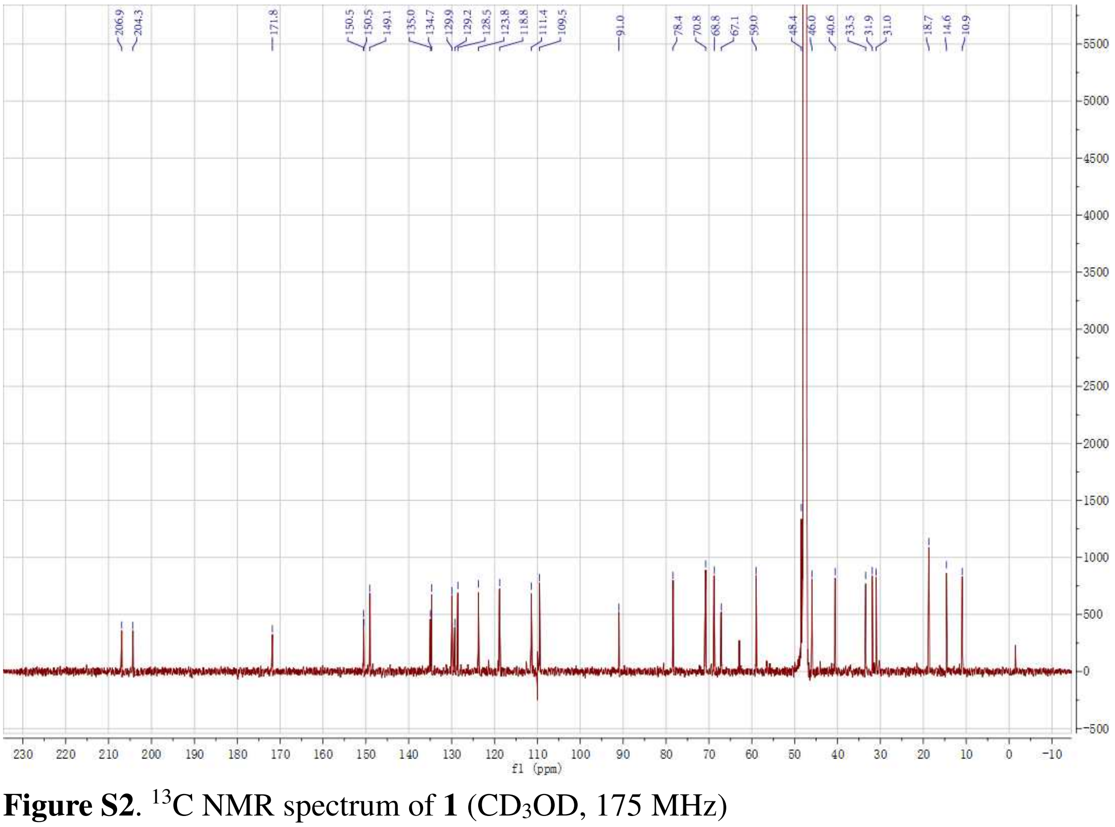

Figure S2. ^{13}C NMR spectrum of **1** (CD_3OD, 175 MHz)

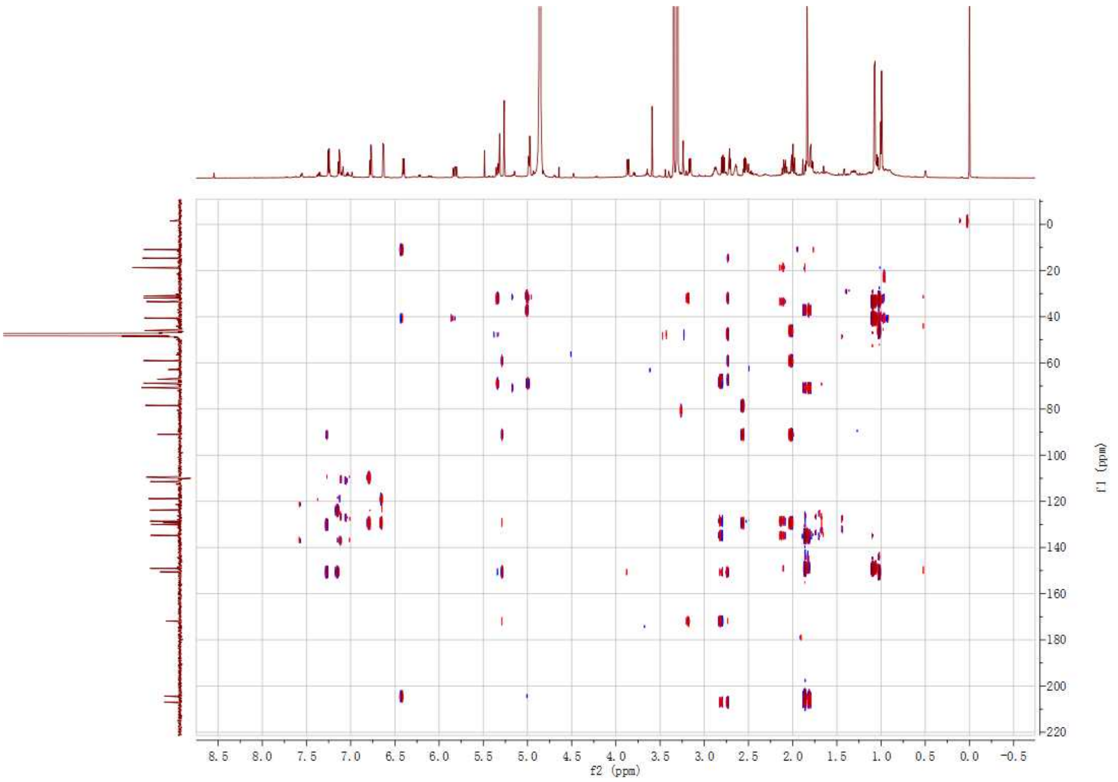

Figure S3. HSQC spectrum of **1** (CD_3OD)

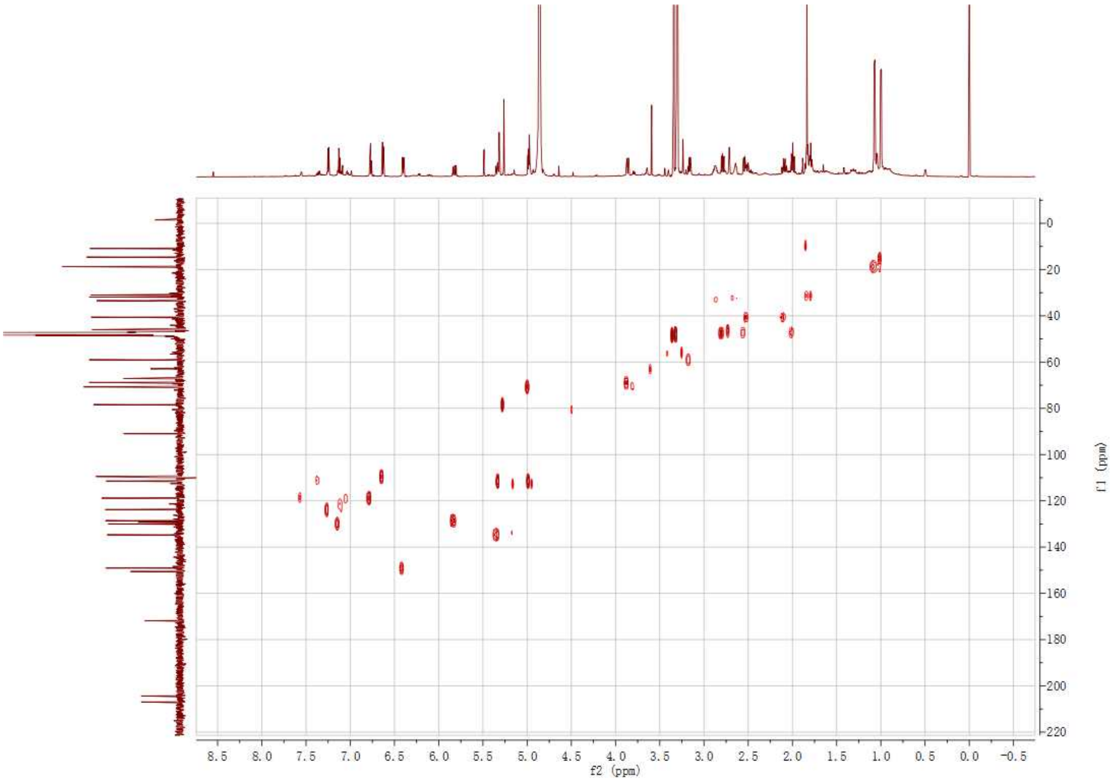

Figure S4. HMBC spectrum of **1** (CD_3OD)

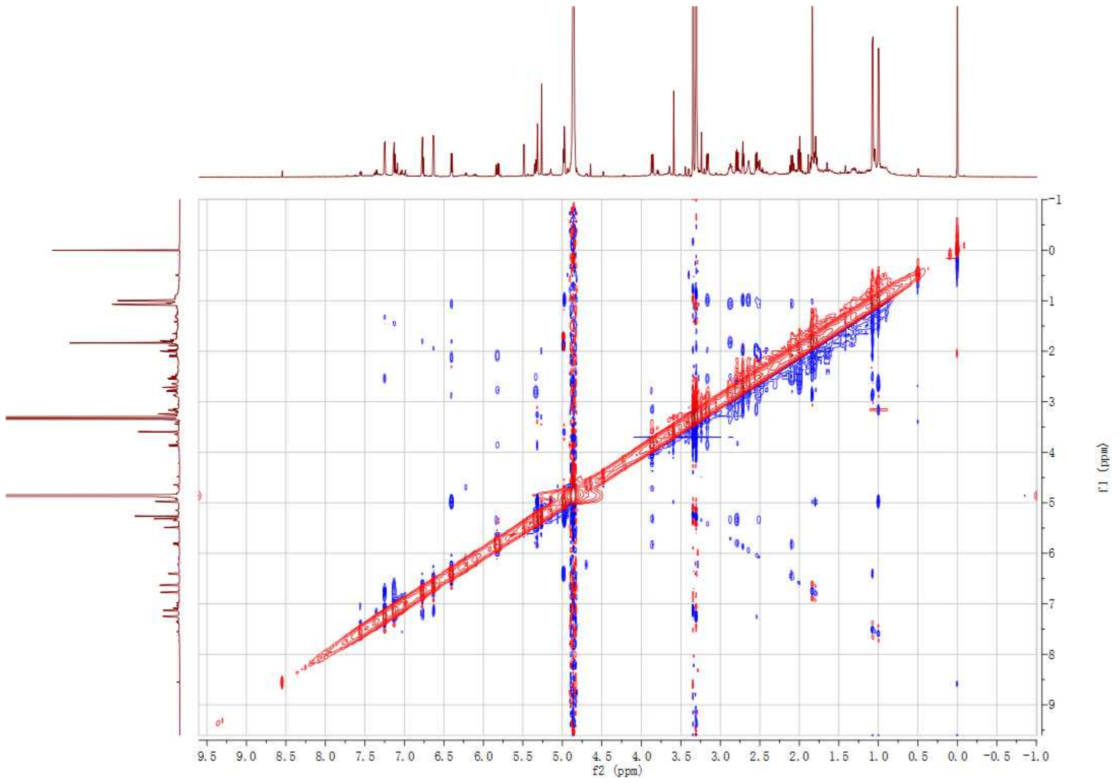

Figure S5. NOESY spectrum of **1** (CD_3OD)

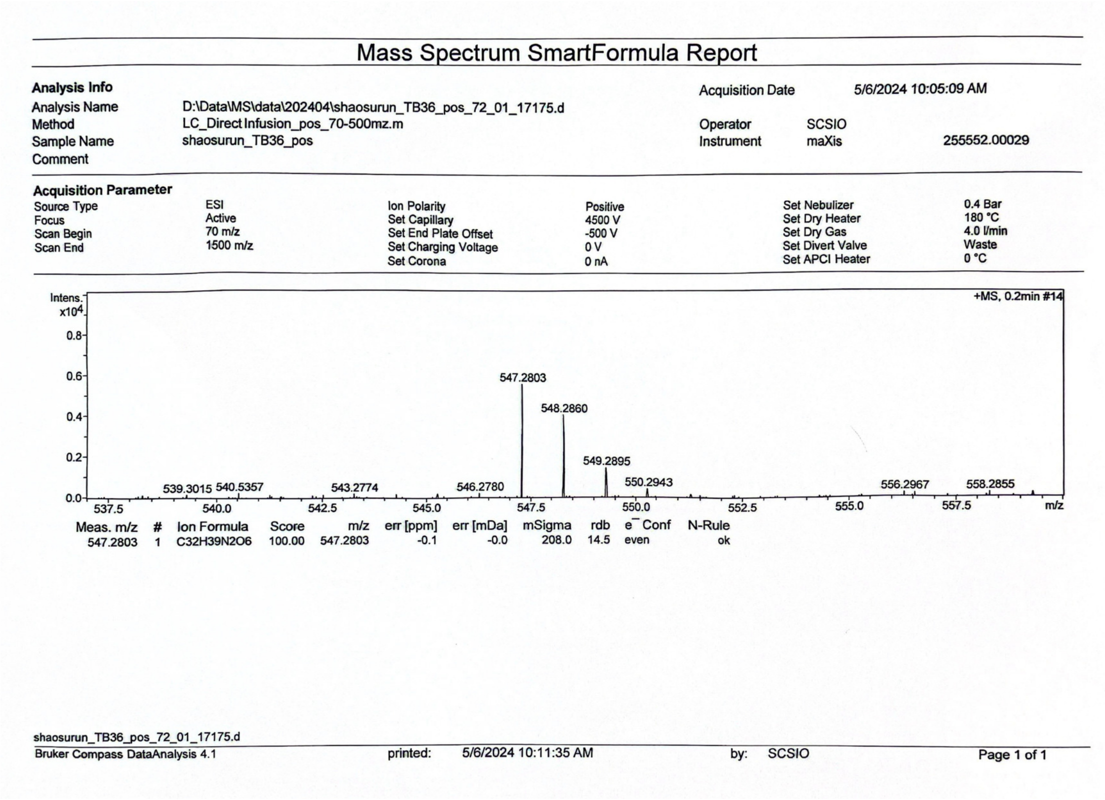

Figure S6. HRESIMS spectrum of **1**

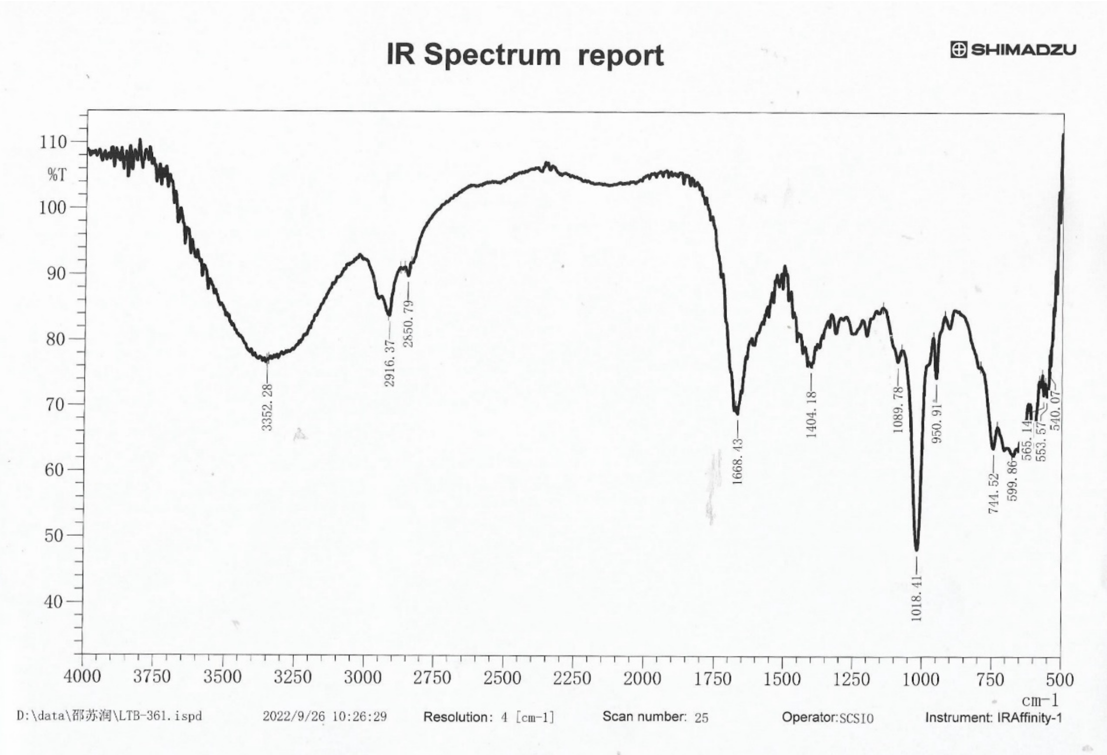

Figure S7. IR spectrum of **1**

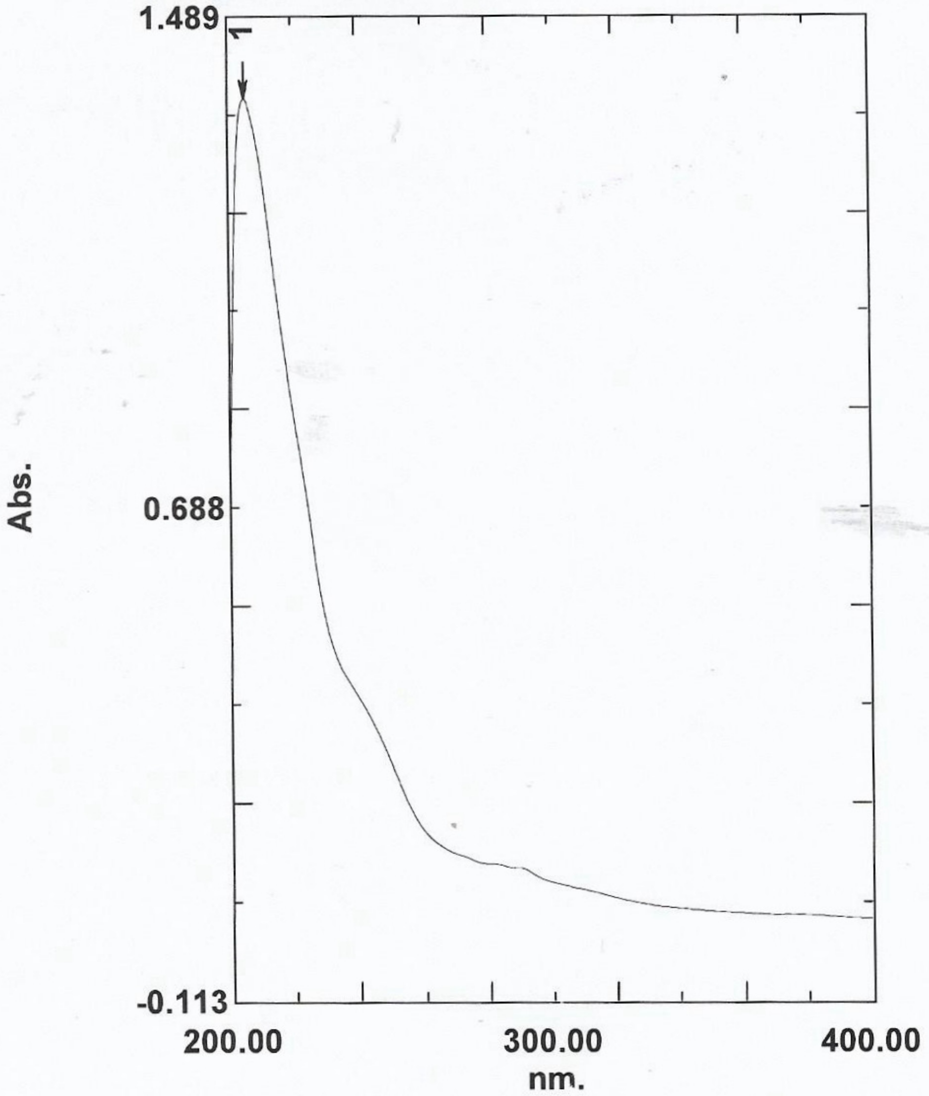

Wavelength （nm）

No.	P/V	波长(nm)	Abs.	描述
1	⬆	205. 20	1. 355	

Figure S8. UV spectrum of **1**

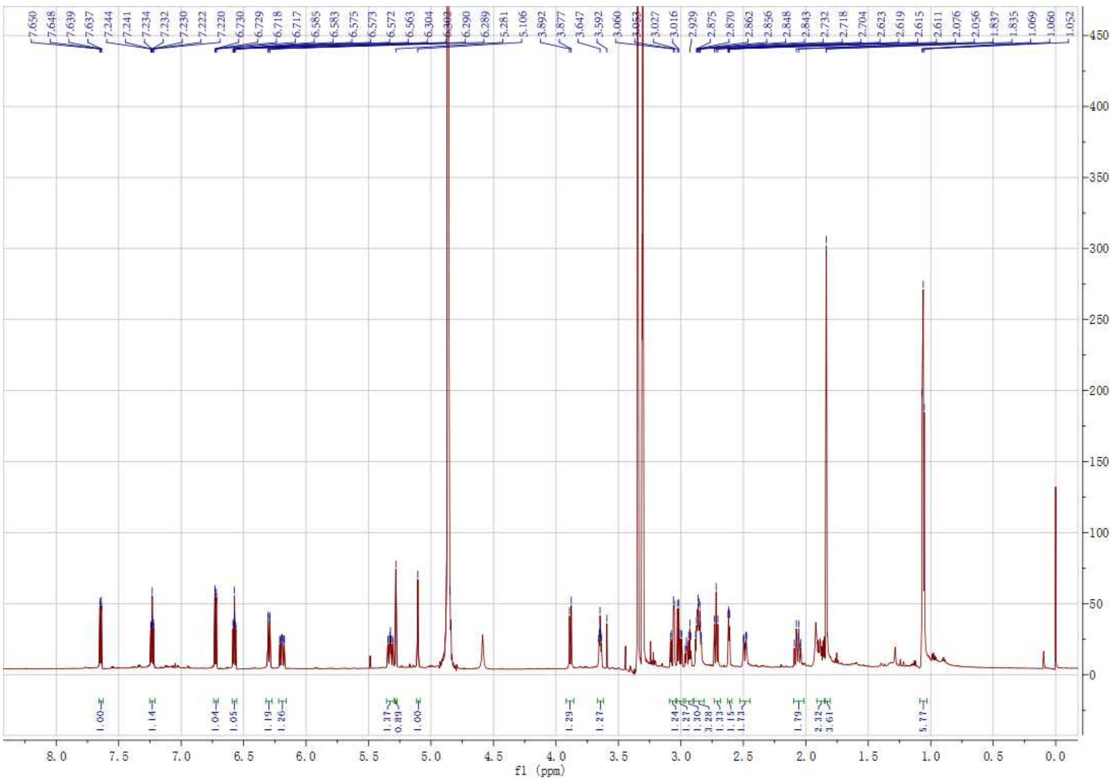

Figure S9. ^{1}H NMR spectrum of **2** (CD_3OD, 700 MHz)

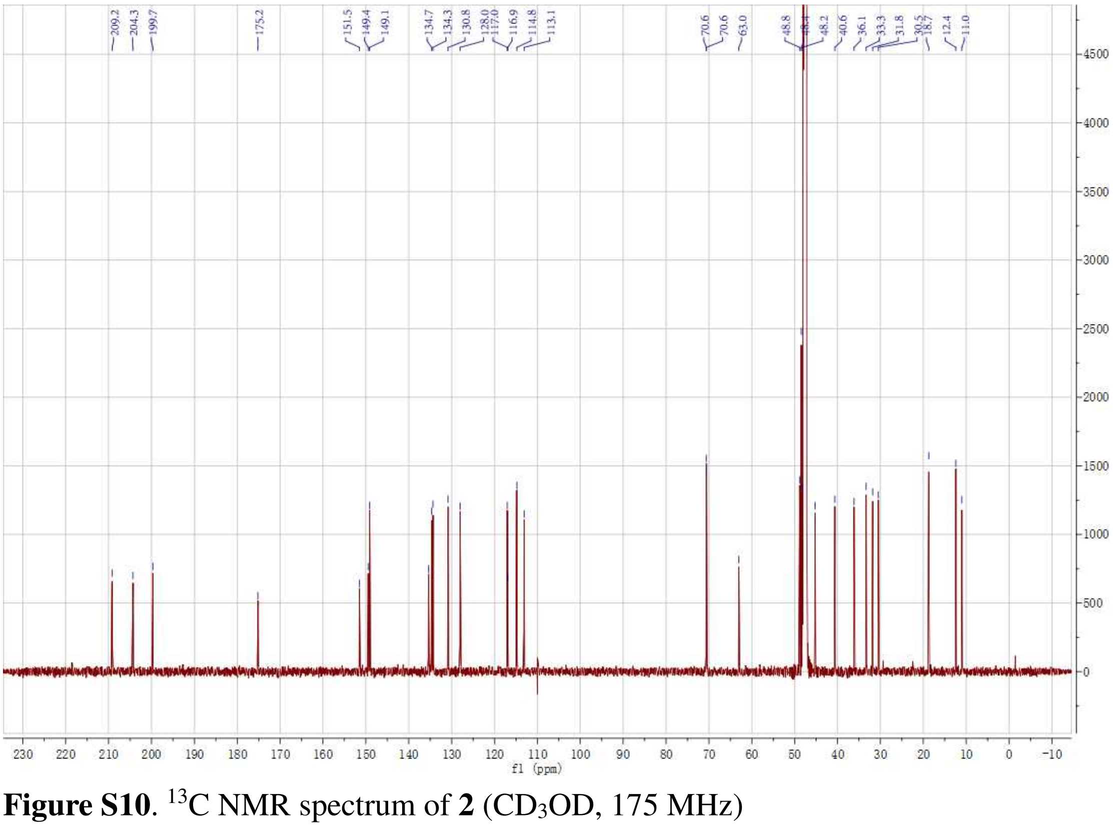

Figure S10. ^{13}C NMR spectrum of **2** (CD_3OD, 175 MHz)

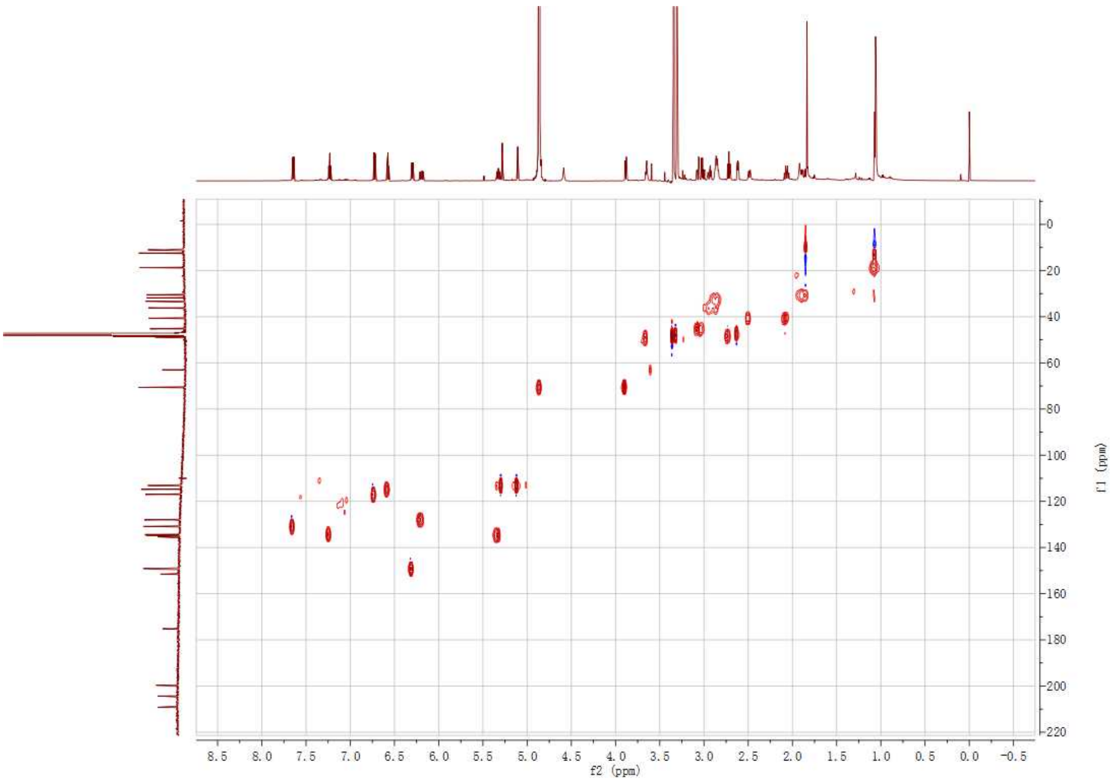

Figure S11. HSQC spectrum of **2** (CD_3OD)

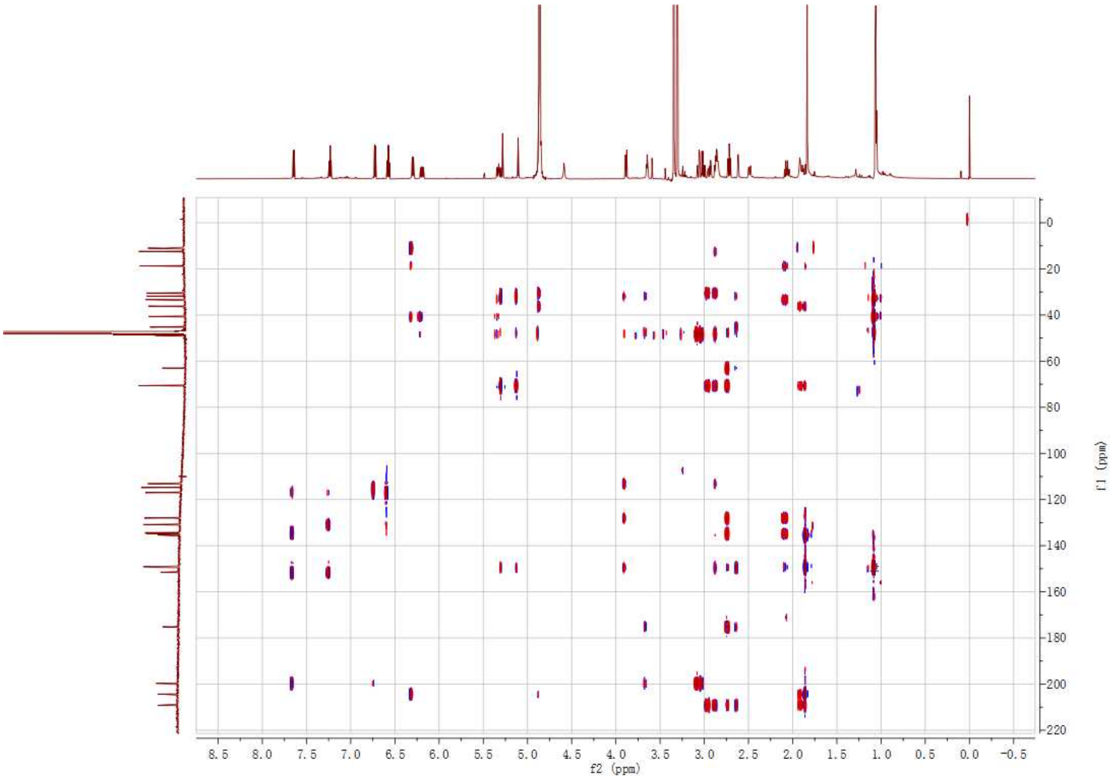

Figure S12. HMBC spectrum of **2** (CD_3OD)

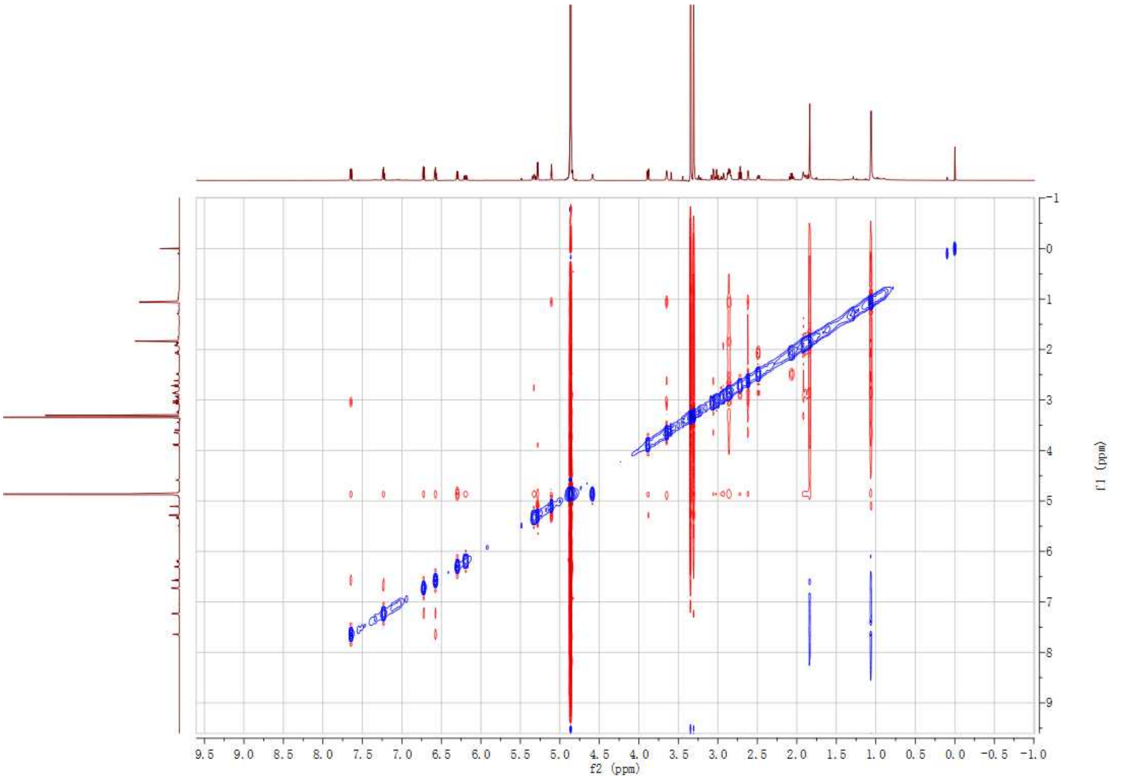

Figure S13. NOESY spectrum of **2** (CD_3OD)

Mass Spectrum SmartFormula Report

Analysis Info

Analysis Name	D:\Data\MS\data\202109\shaosurun_TB37_pos_2_01_11599.d	Acquisition Date	9/28/2021 10:33:23 AM
Method	LC_Direct Infusion_pos_70-500mz.m	Operator	SCSIO
Sample Name	shaosurun_TB37_pos	Instrument	maXis 255552.00029
Comment			

Acquisition Parameter

Source Type	ESI	Ion Polarity	Positive	Set Nebulizer	0.4 Bar
Focus	Active	Set Capillary	4500 V	Set Dry Heater	180 °C
Scan Begin	70 m/z	Set End Plate Offset	-500 V	Set Dry Gas	4.0 l/min
Scan End	1500 m/z	Set Charging Voltage	0 V	Set Divert Valve	Waste
		Set Corona	0 nA	Set APCI Heater	0 °C

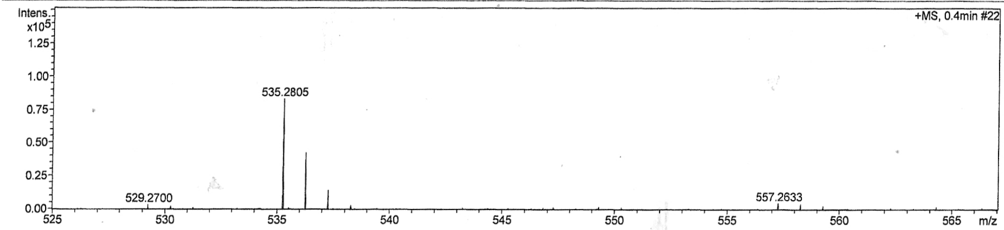

Meas. m/z	#	Ion Formula	Score	m/z	err [ppm]	err [mDa]	mSigma	rdb	e⁻ Conf	N-Rule
535.2805	1	C31H39N2O6	100.00	535.2803	0.4	0.2	92.6	13.5	even	ok
557.2633	1	C31H38N2NaO6	100.00	557.2622	1.9	1.1	173.9	13.5	even	ok

shaosurun_TB37_pos_2_01_11599.d
Bruker Compass DataAnalysis 4.1 printed: 9/28/2021 10:41:32 AM by: SCSIO Page 1 of 1

Figure S14. HRESIMS spectrum of **2**

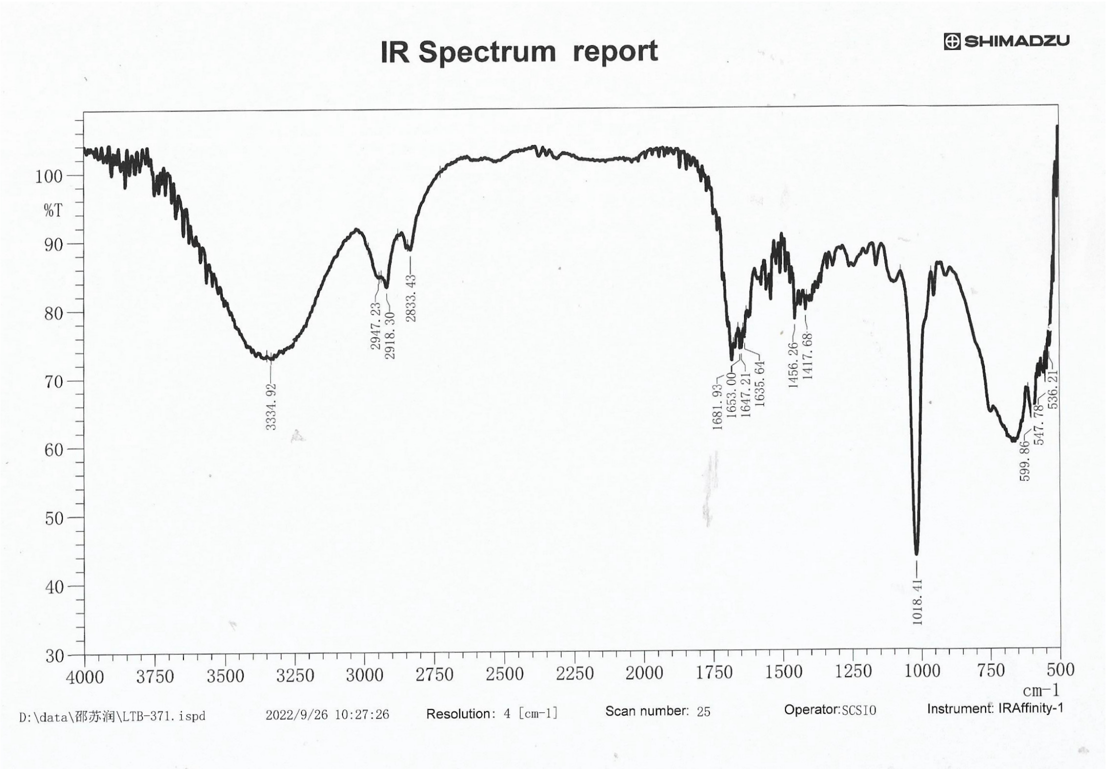

Figure S15. IR spectrum of **2**

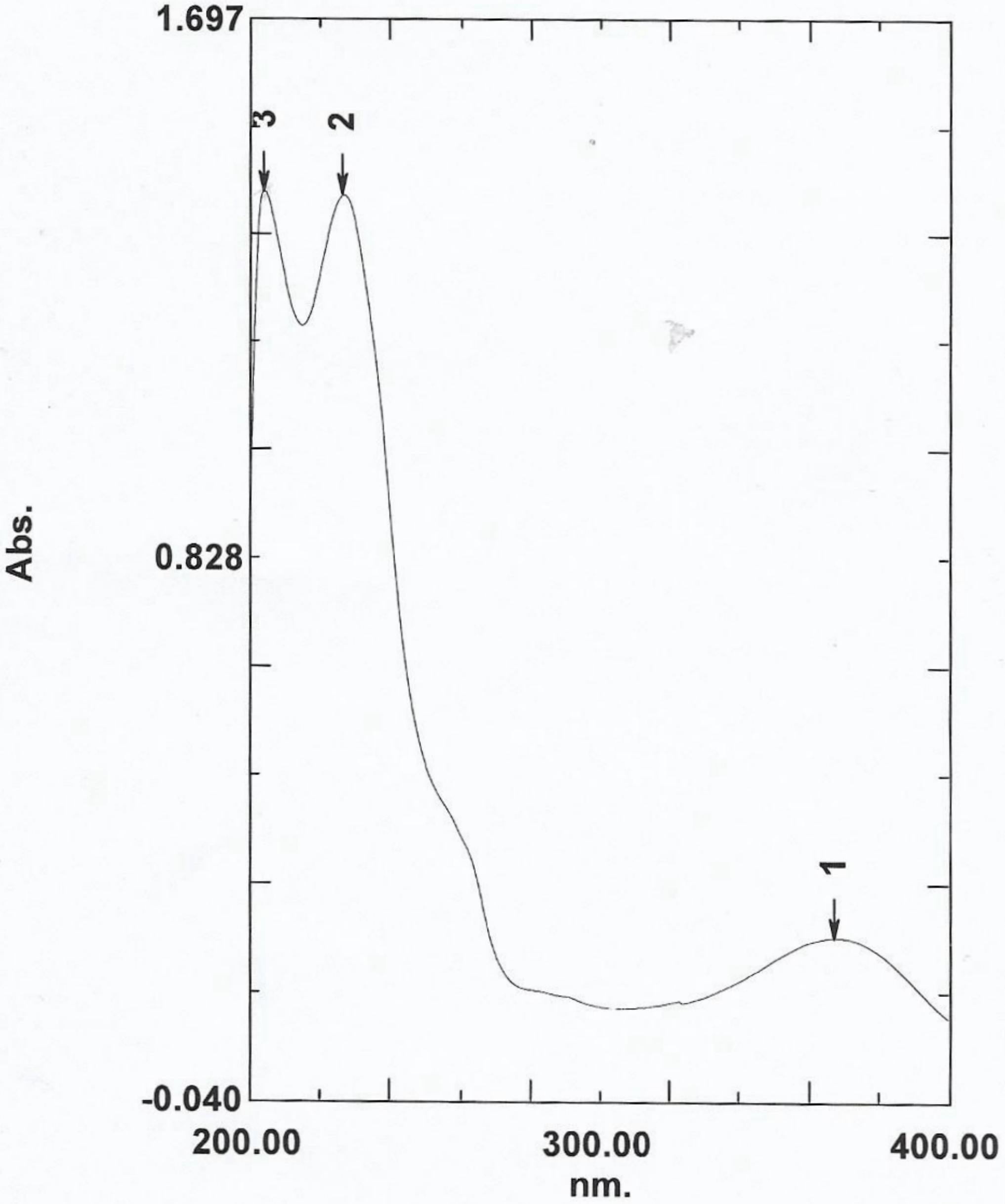

Wavelength （nm）

No.	P/V	波长(nm)	Abs.	描述
1	⬆	367.40	0.222	
2	⬆	226.60	1.413	
3	⬆	203.80	1.419	

Figure S16. UV spectrum of **2**

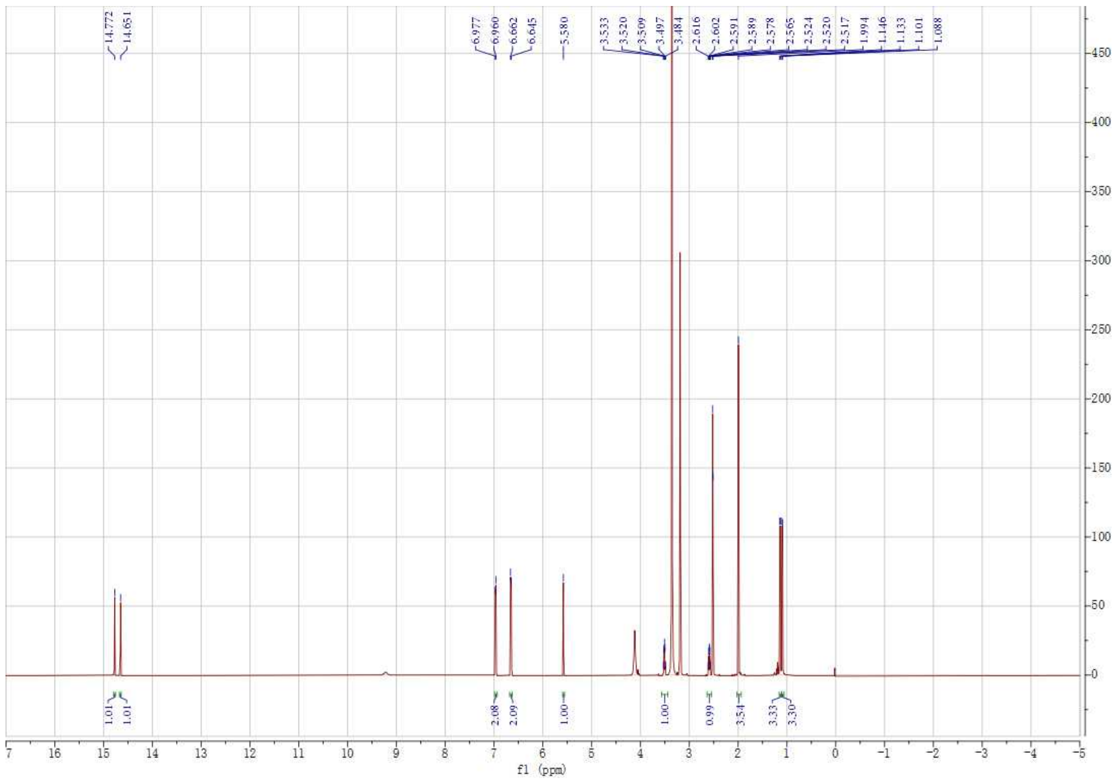

Figure S17. ^{1}H NMR spectrum of **4** (DMSO-d_6, 500 MHz)

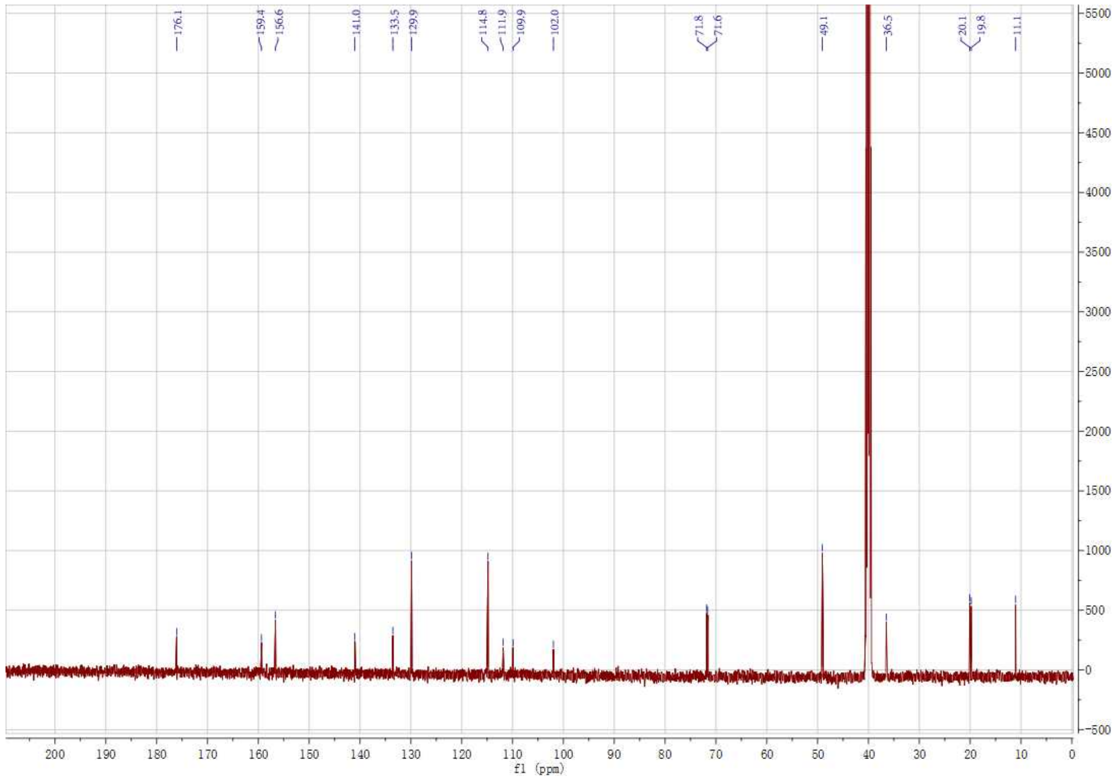

Figure S18. ^{13}C NMR spectrum of **4** (DMSO-d_6, 125 MHz)

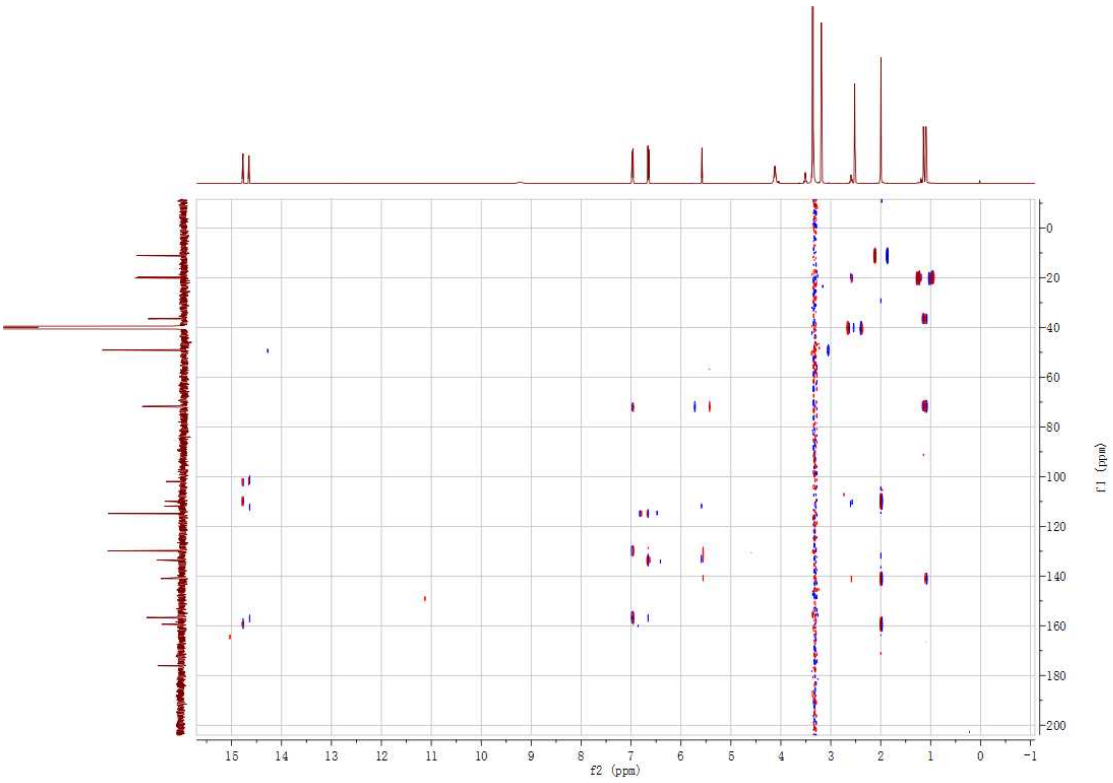

Figure S19. HSQC spectrum of **4** (DMSO-d_6)

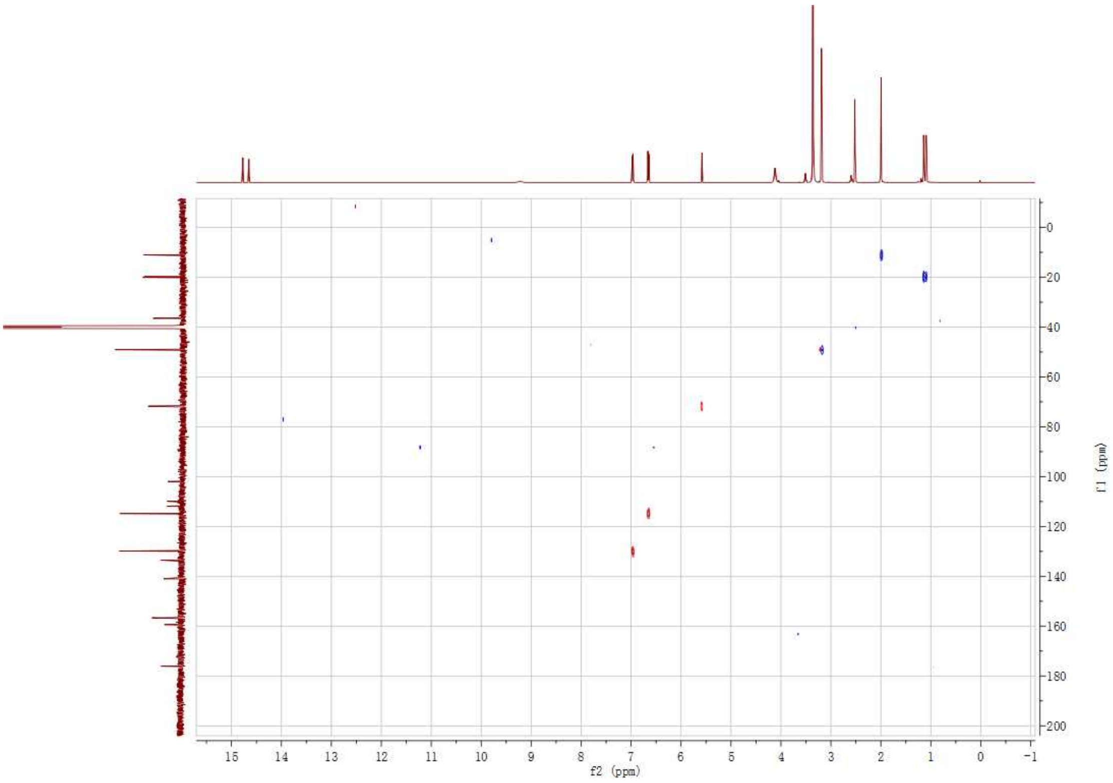

Figure S20. HMBC spectrum of **4** (DMSO-d_6)

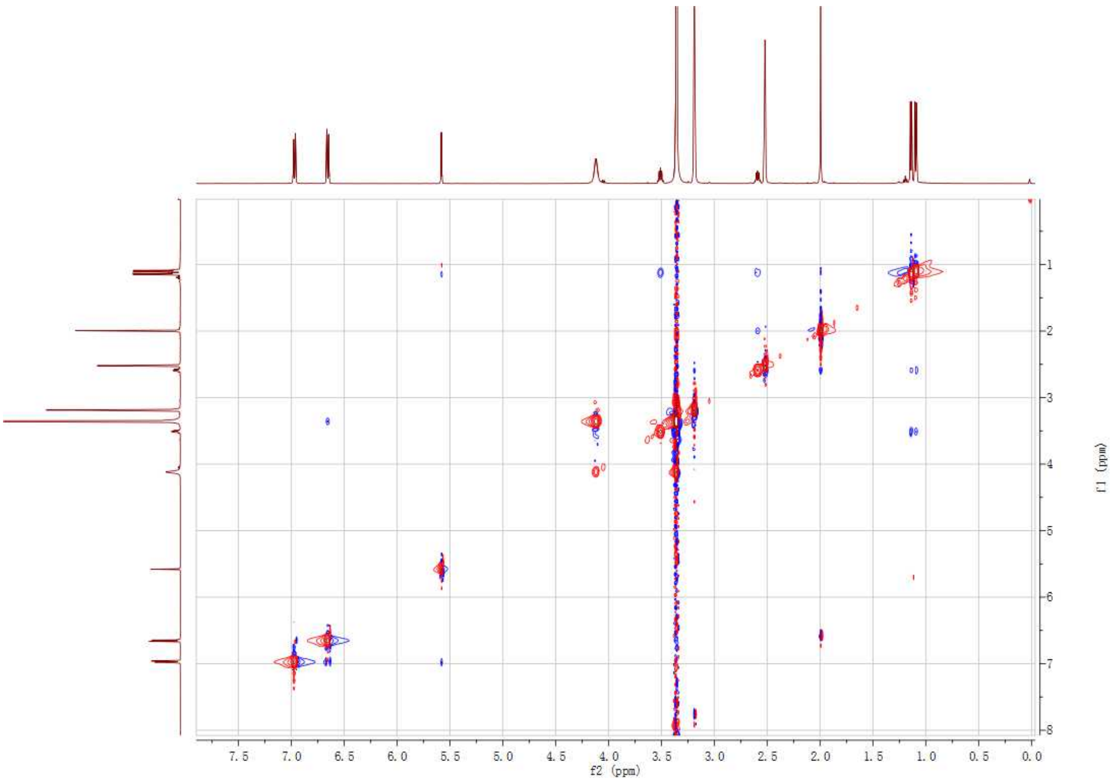

Figure S21. NOESY spectrum of **4** (DMSO-d_6)

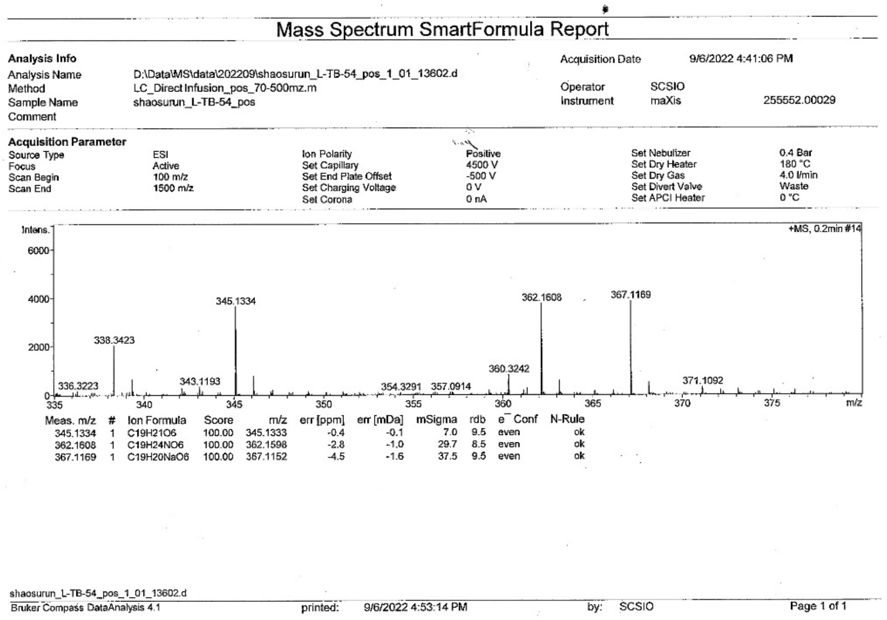

Mass Spectrum SmartFormula Report

**Analysis Info**		Acquisition Date	9/6/2022 4:41:06 PM	
Analysis Name	D:\Data\MS\data\202209\shaosurun_L-TB-54_pos_1_01_13602.d			
Method	LC_Direct Infusion_pos_70-500mz.m	Operator	SCSIO	
Sample Name	shaosurun_L-TB-54_pos	Instrument	maXis	255552.00029
Comment				

**Acquisition Parameter**					
Source Type	ESI	Ion Polarity	Positive	Set Nebulizer	0.4 Bar
Focus	Active	Set Capillary	4500 V	Set Dry Heater	180 °C
Scan Begin	100 m/z	Set End Plate Offset	-500 V	Set Dry Gas	4.0 l/min
Scan End	1500 m/z	Set Charging Voltage	0 V	Set Divert Valve	Waste
		Set Corona	0 nA	Set APCI Heater	0 °C

Meas. m/z	#	Ion Formula	Score	m/z	err [ppm]	err [mDa]	mSigma	rdb	e¯ Conf	N-Rule
345.1334	1	C19H21O6	100.00	345.1333	-0.4	-0.1	7.0	9.5	even	ok
362.1608	1	C19H24NO6	100.00	362.1598	-2.8	-1.0	29.7	8.5	even	ok
367.1169	1	C19H20NaO6	100.00	367.1152	-4.5	-1.6	37.5	9.5	even	ok

shaosurun_L-TB-54_pos_1_01_13602.d
Bruker Compass DataAnalysis 4.1 printed: 9/6/2022 4:53:14 PM by: SCSIO Page 1 of 1

Figure S22. HRESIMS spectrum of **4**

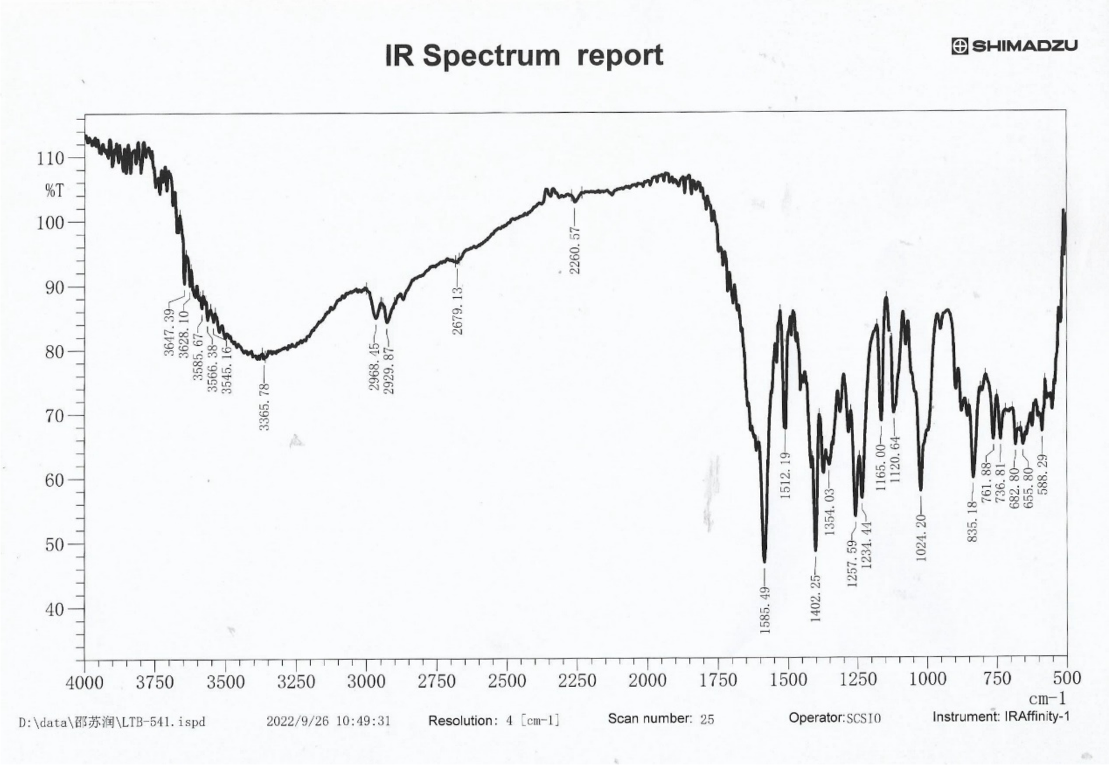

Figure S23. IR spectrum of **4**

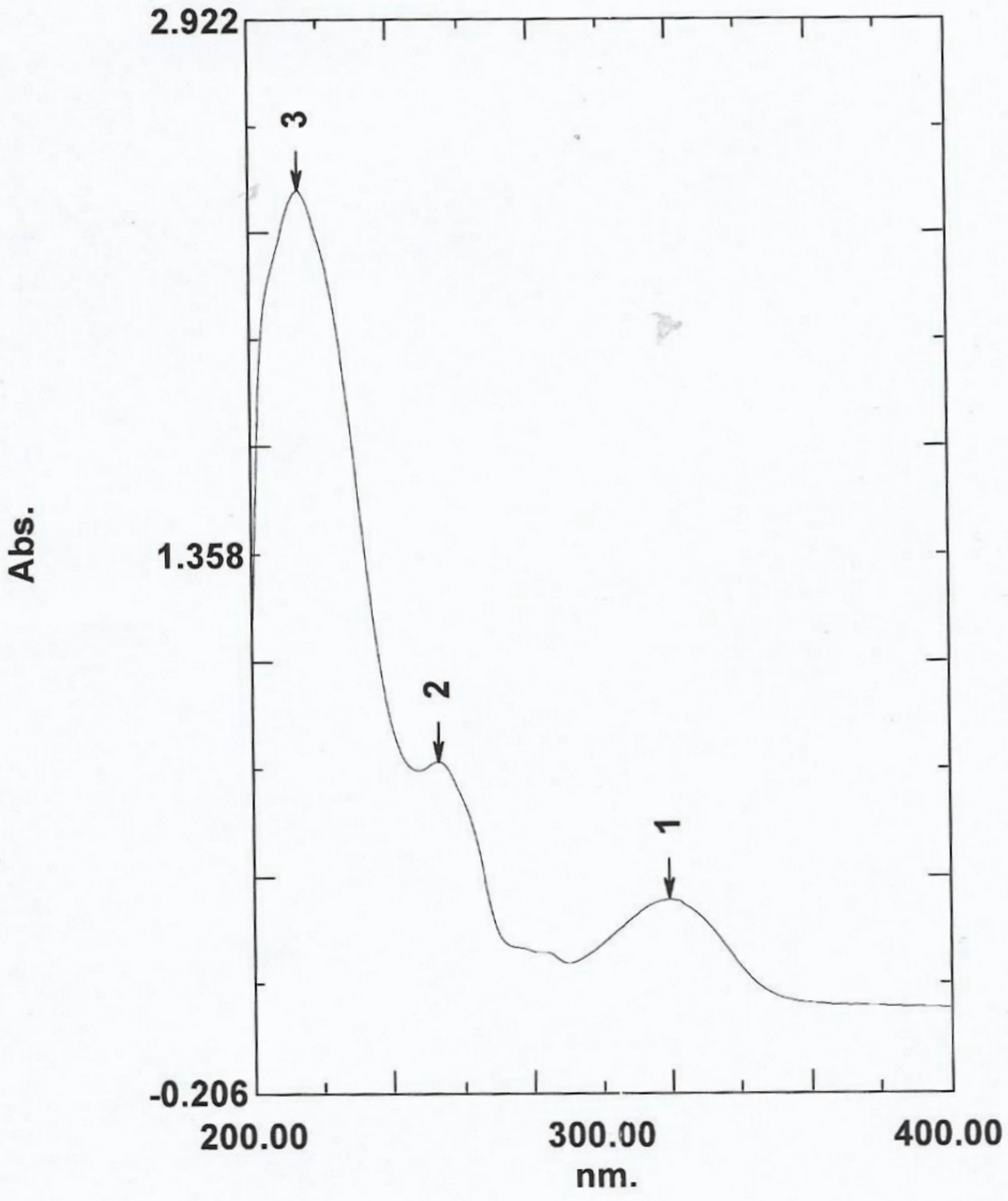

Wavelength （nm）

No.	P/V	波长(nm)	Abs.	描述
1		319.20	0.352	
2		253.00	0.753	
3		214.00	2.421	

Figure S24. UV spectrum of **4**

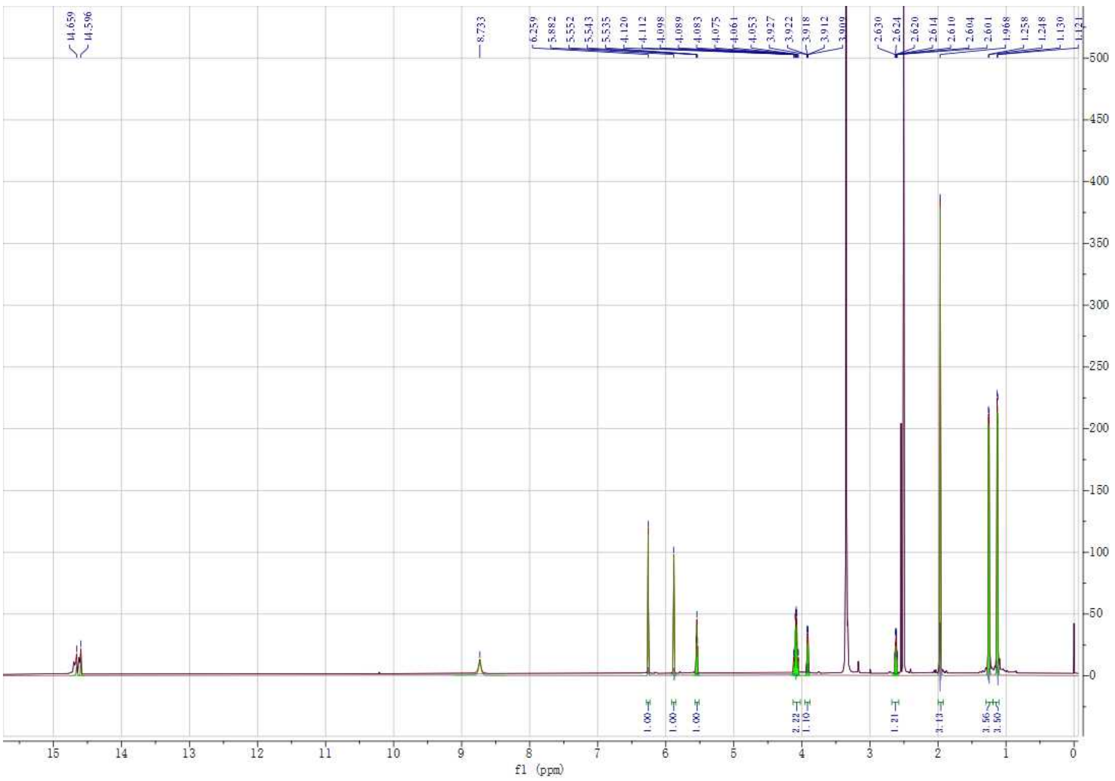

Figure S25. ^{1}H NMR spectrum of **5** (DMSO-d_6, 700 MHz)

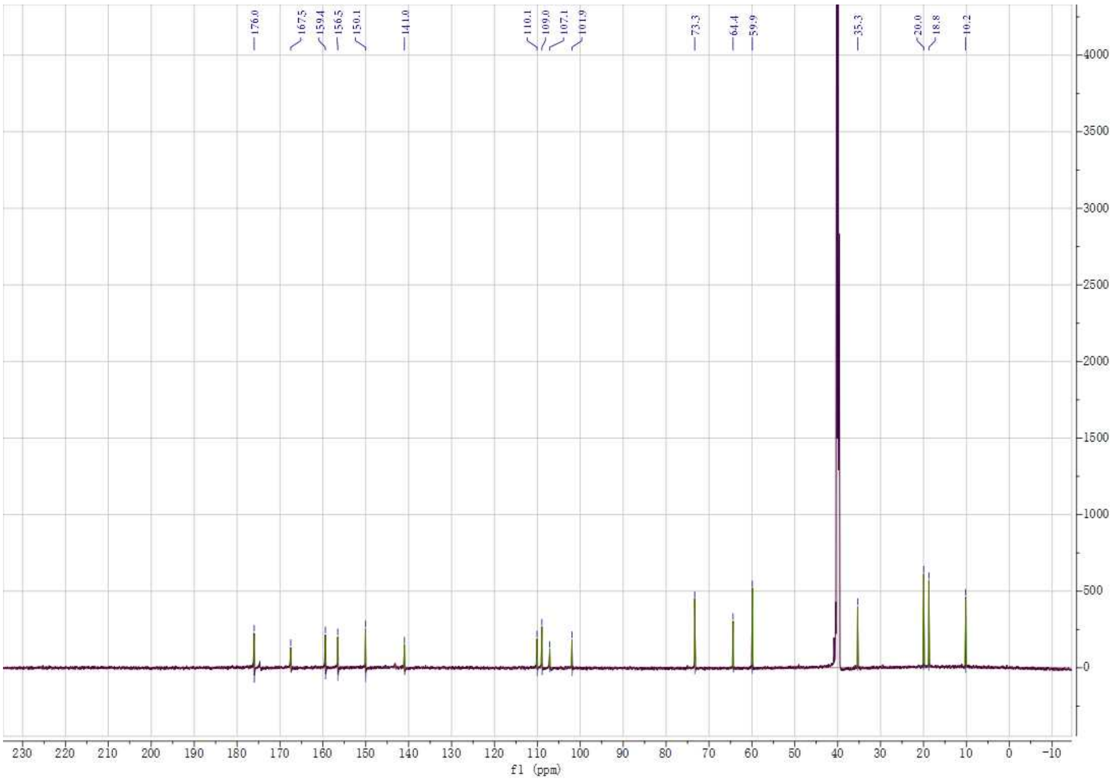

Figure S26. ^{13}C NMR spectrum of **5** (DMSO-d_6, 175 MHz)

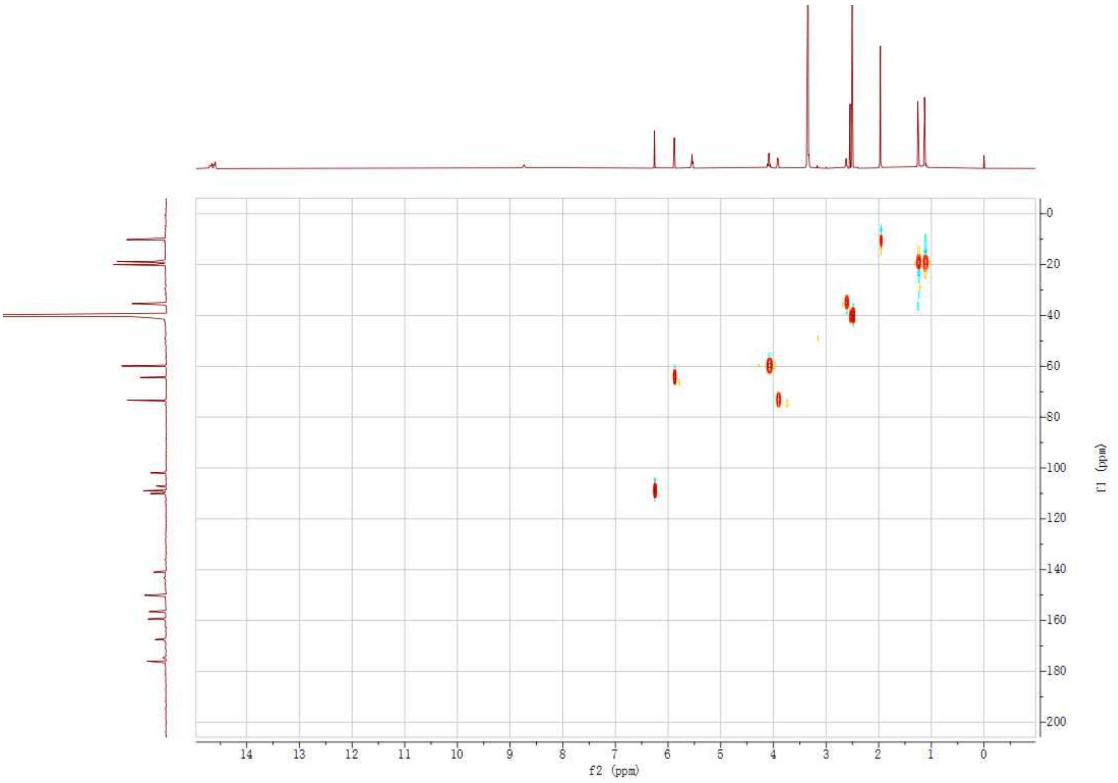

Figure S27. HSQC spectrum of **5** (DMSO-d_6)

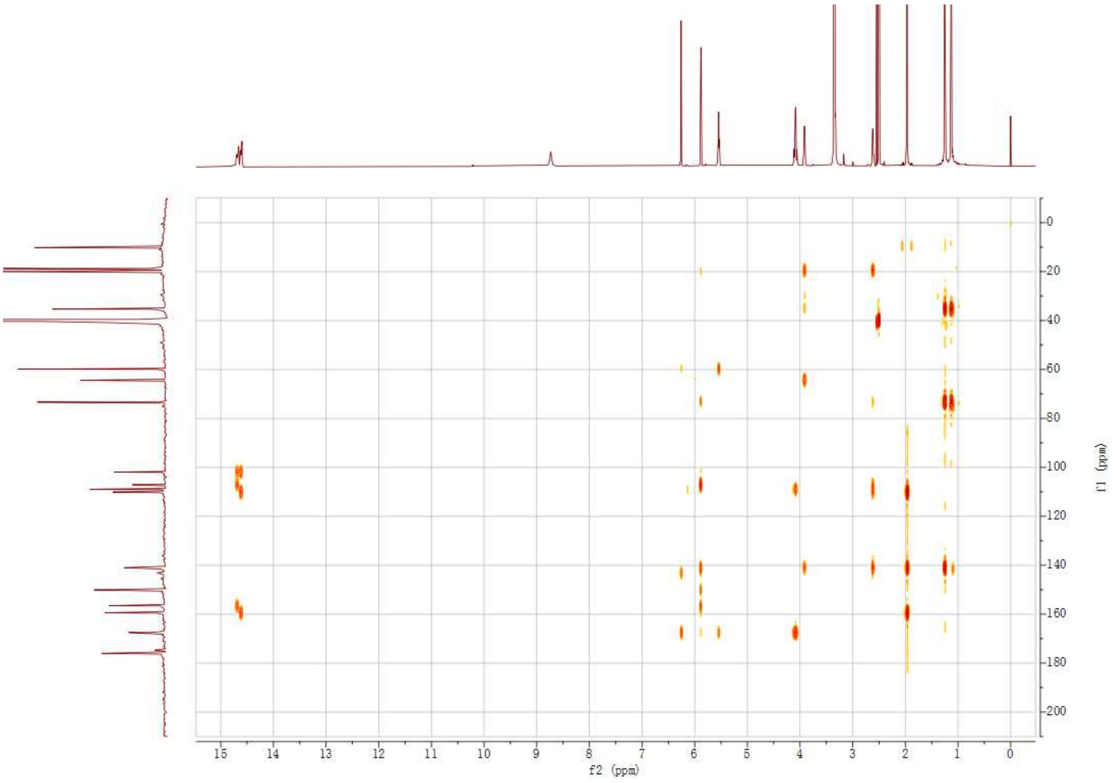

Figure S28. HMBC spectrum of **5**(DMSO-d_6)

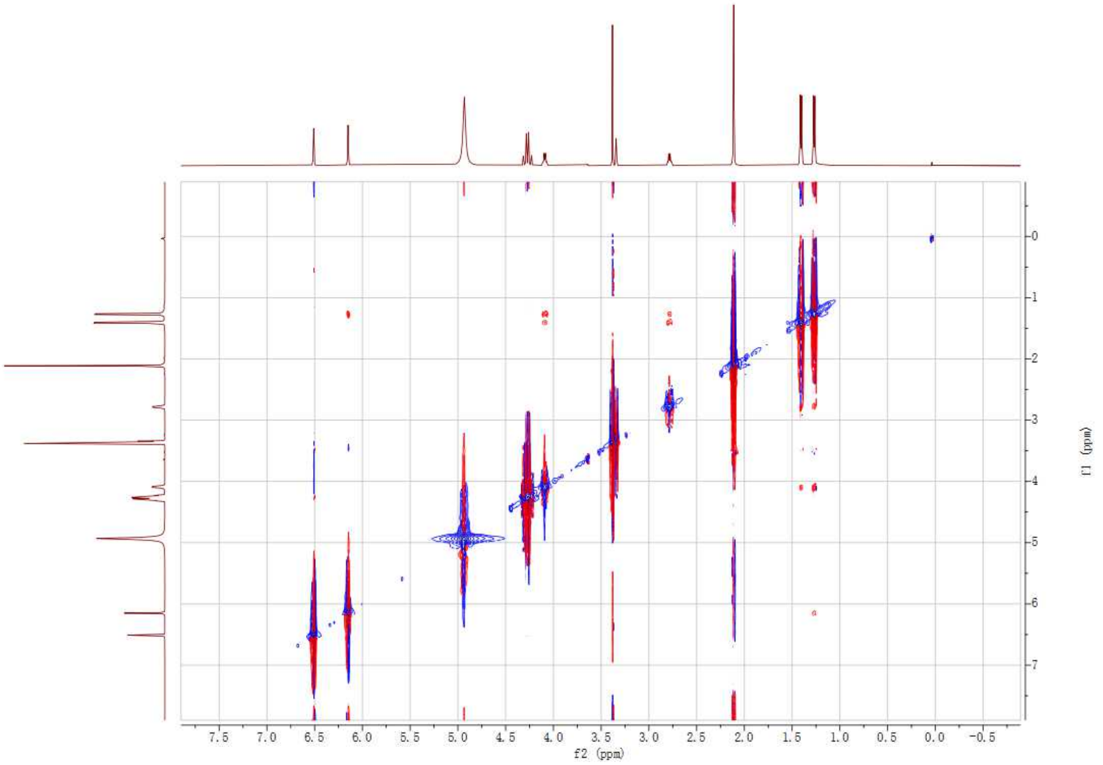

Figure S29. NOESY spectrum of **5** (CD_3OD)

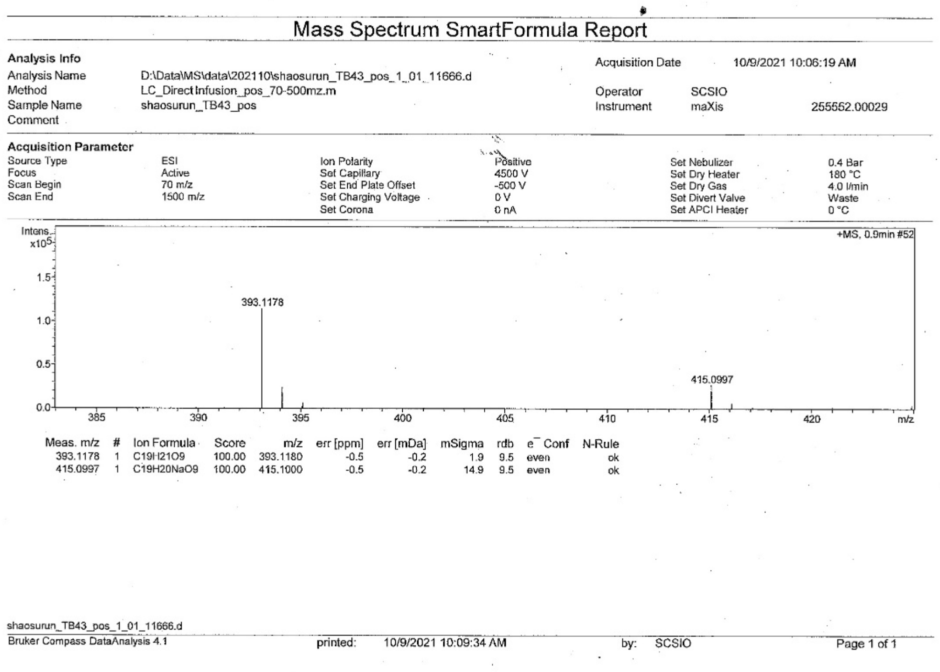

Figure S30. HRESIMS spectrum of **5**

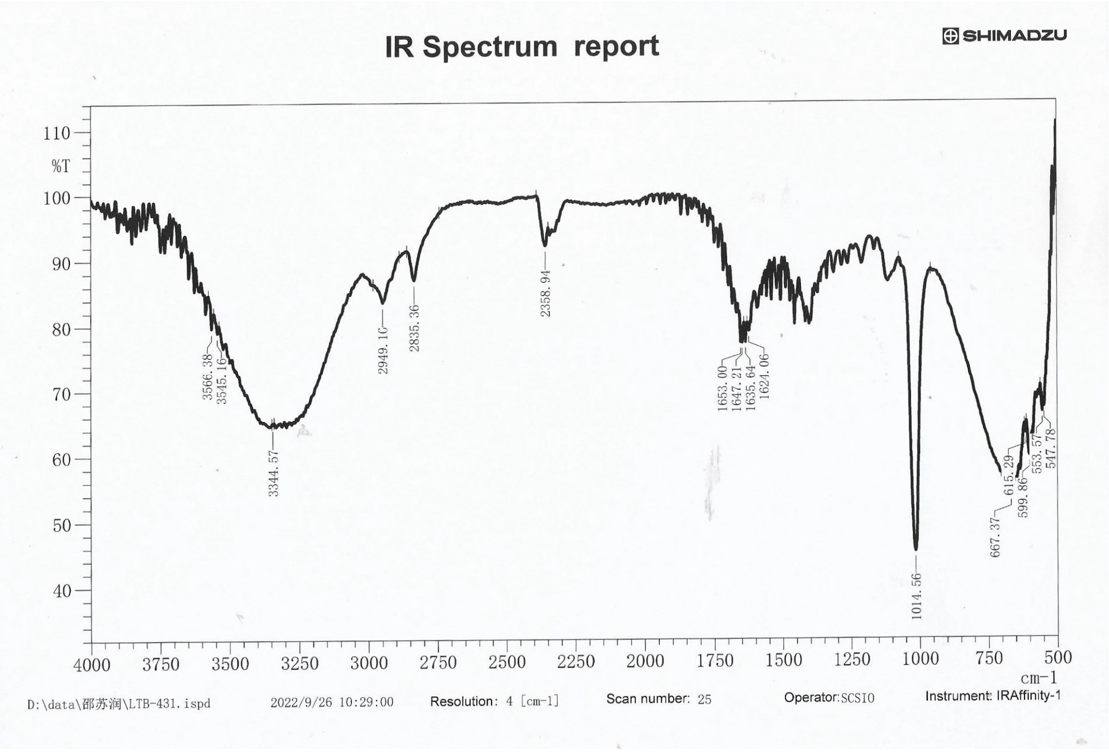

Figure S31. IR spectrum of **5**

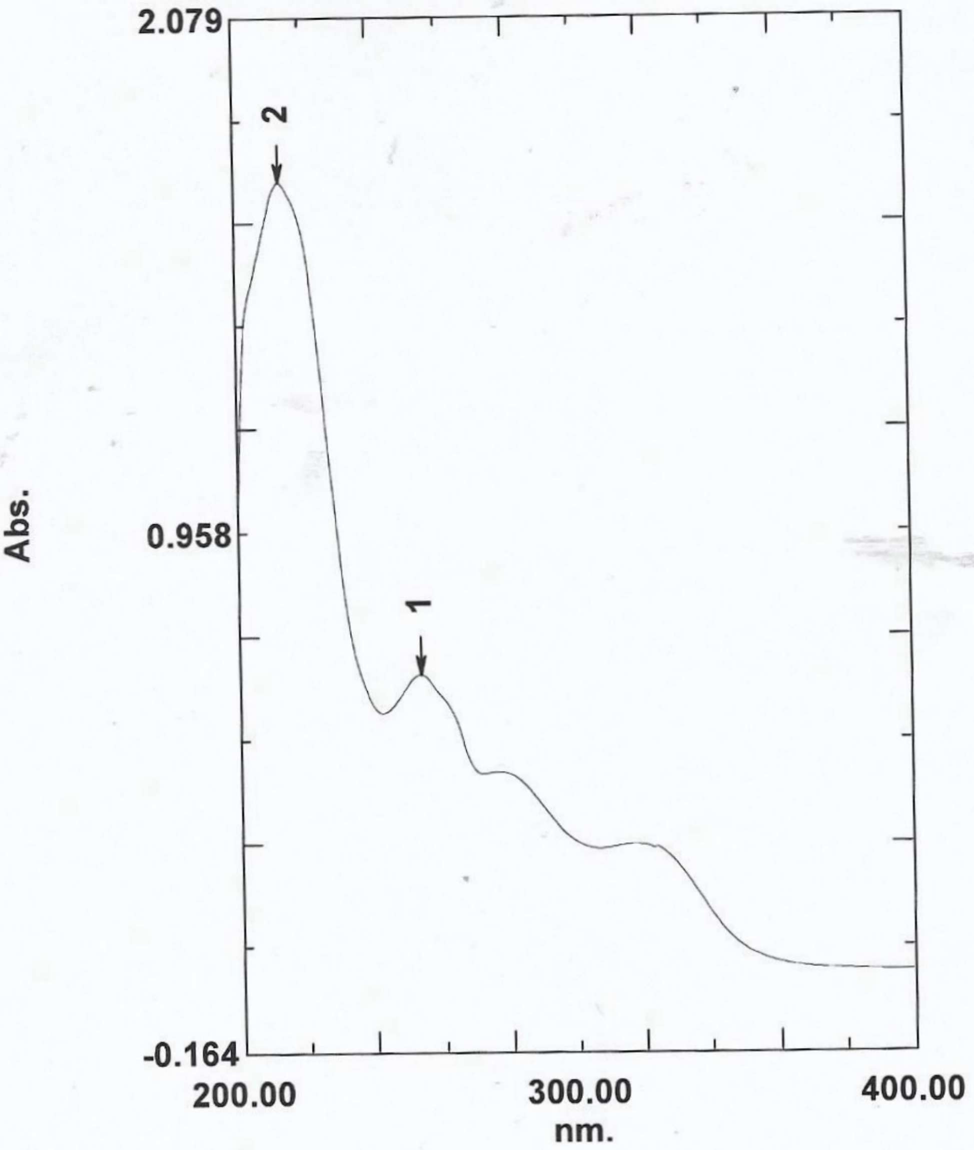

Wavelength （nm）

No.	P/V	波长(nm)	Abs.	描述
1	⬆	253.60	0.651	
2	⬆	213.20	1.720	

Figure S32. UV spectrum of **5**

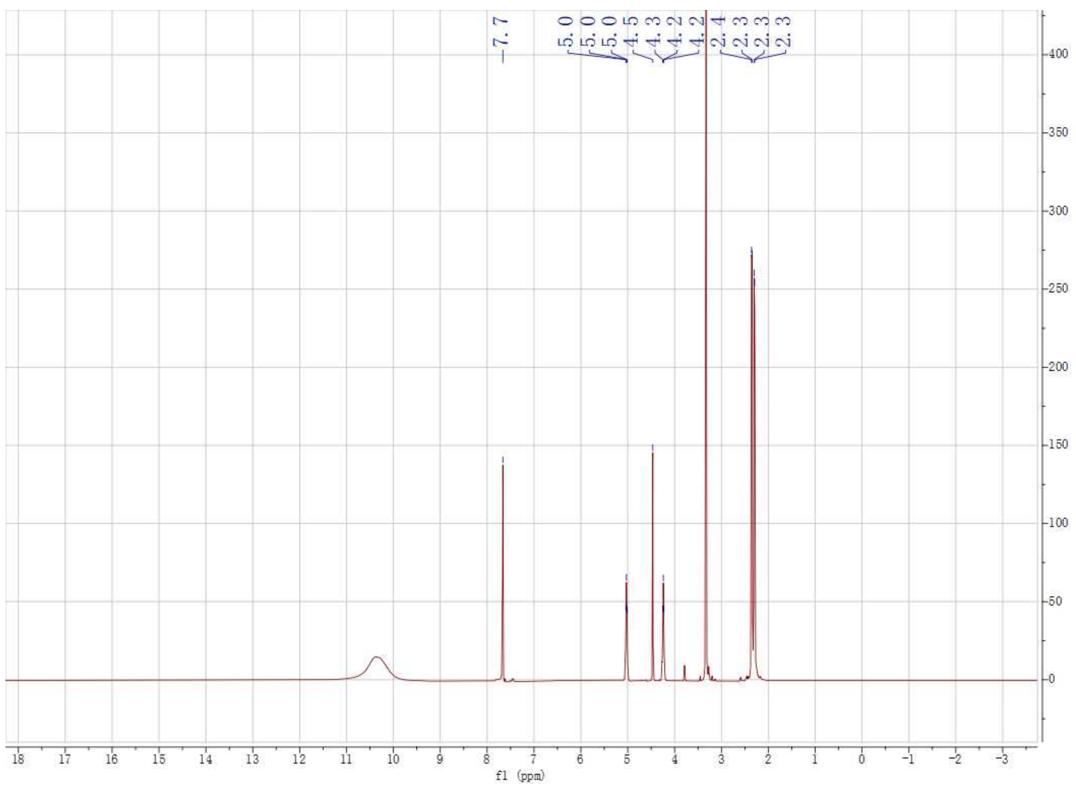

Figure S33. ^{1}H NMR spectrum of **7** (DMSO-d_6, 700 MHz)

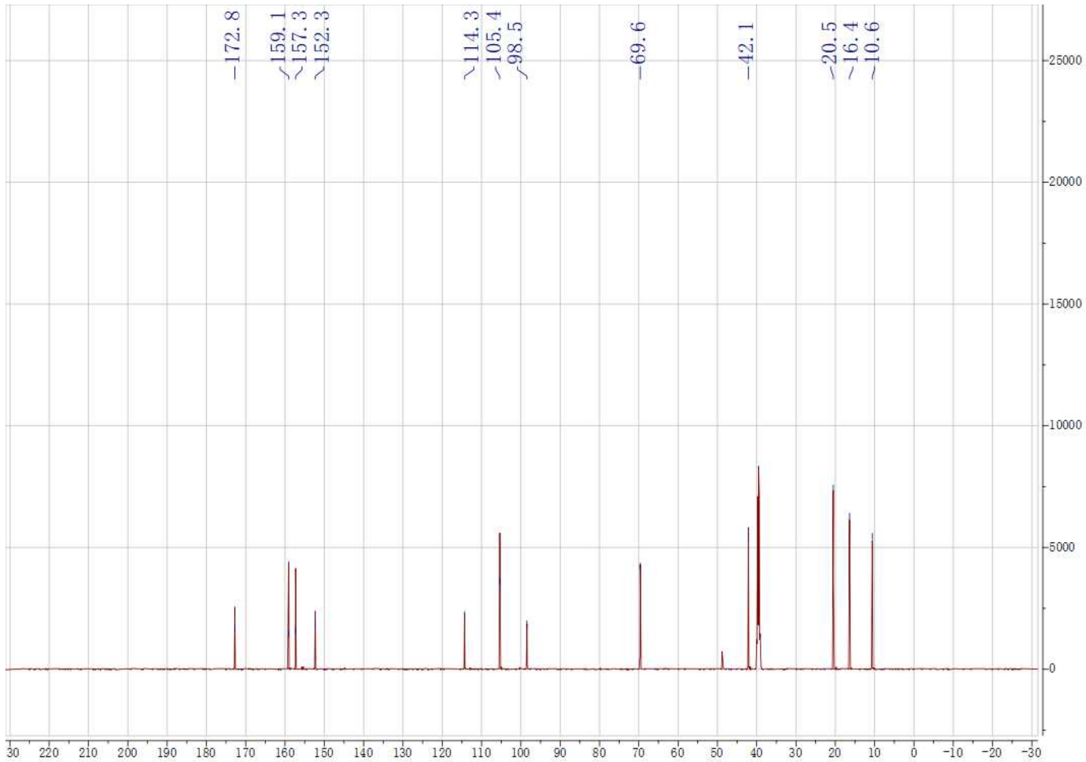

Figure S34. ^{13}C NMR spectrum of **7** (DMSO-d_6, 175 MHz)

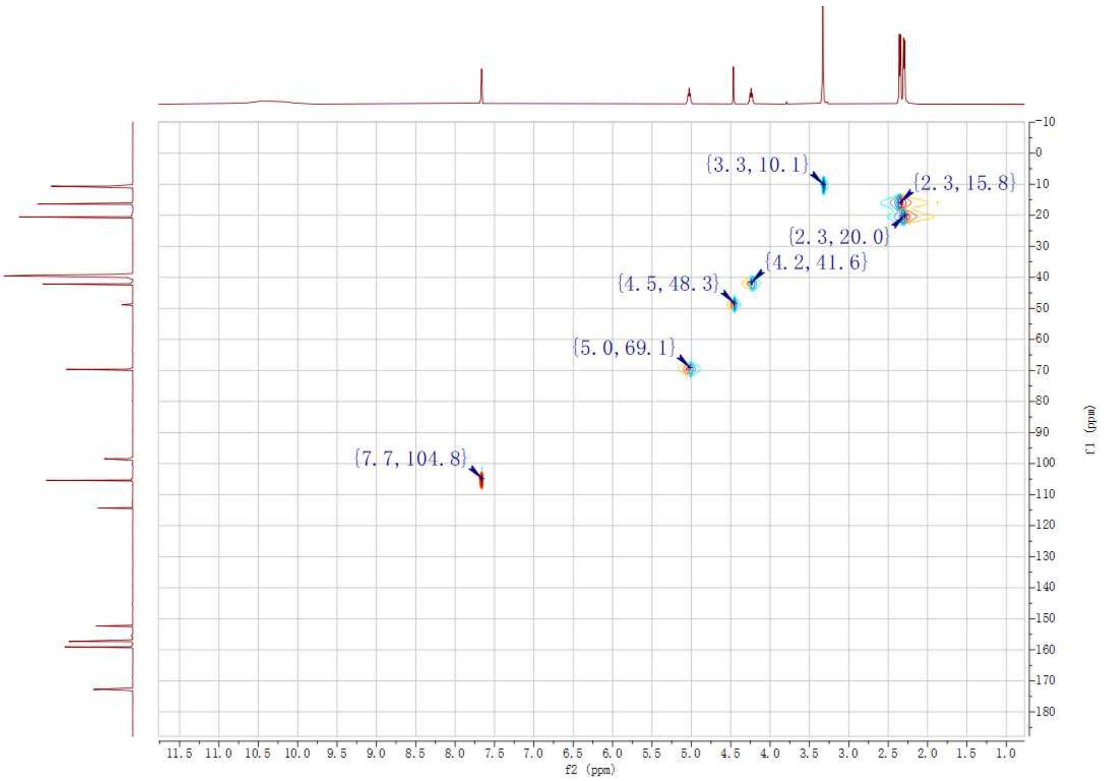

Figure S35. HSQC spectrum of **7** (DMSO-d_6)

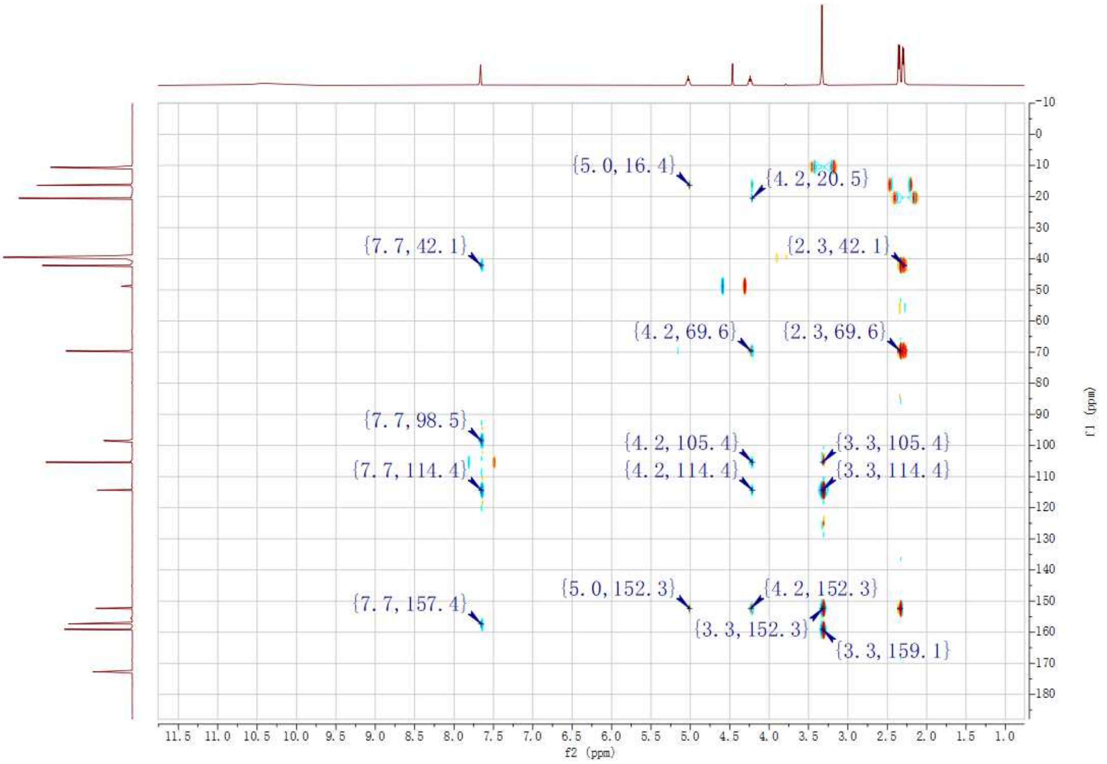

Figure S36. HMBC spectrum of **7** (DMSO-d_6)

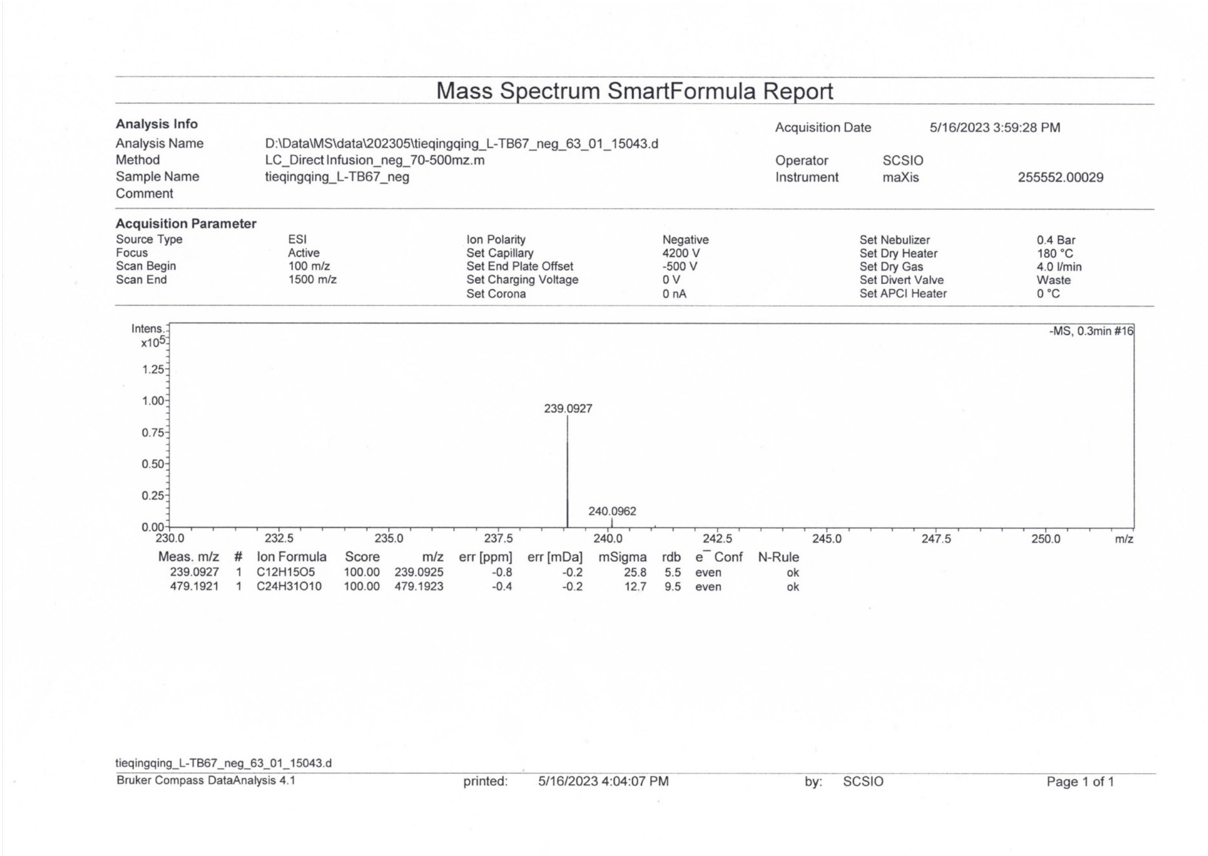

Mass Spectrum SmartFormula Report

Analysis Info

Analysis Name	D:\Data\MS\data\202305\tieqingqing_L-TB67_neg_63_01_15043.d	Acquisition Date	5/16/2023 3:59:28 PM
Method	LC_Direct Infusion_neg_70-500mz.m	Operator	SCSIO
Sample Name	tieqingqing_L-TB67_neg	Instrument	maXis 255552.00029
Comment			

Acquisition Parameter

Source Type	ESI	Ion Polarity	Negative	Set Nebulizer	0.4 Bar
Focus	Active	Set Capillary	4200 V	Set Dry Heater	180 °C
Scan Begin	100 m/z	Set End Plate Offset	-500 V	Set Dry Gas	4.0 l/min
Scan End	1500 m/z	Set Charging Voltage	0 V	Set Divert Valve	Waste
		Set Corona	0 nA	Set APCI Heater	0 °C

Meas. m/z	#	Ion Formula	Score	m/z	err [ppm]	err [mDa]	mSigma	rdb	e⁻ Conf	N-Rule
239.0927	1	C12H15O5	100.00	239.0925	-0.8	-0.2	25.8	5.5	even	ok
479.1921	1	C24H31O10	100.00	479.1923	-0.4	-0.2	12.7	9.5	even	ok

tieqingqing_L-TB67_neg_63_01_15043.d
Bruker Compass DataAnalysis 4.1 printed: 5/16/2023 4:04:07 PM by: SCSIO Page 1 of 1

Figure S37. HRESIMS spectrum of **7**

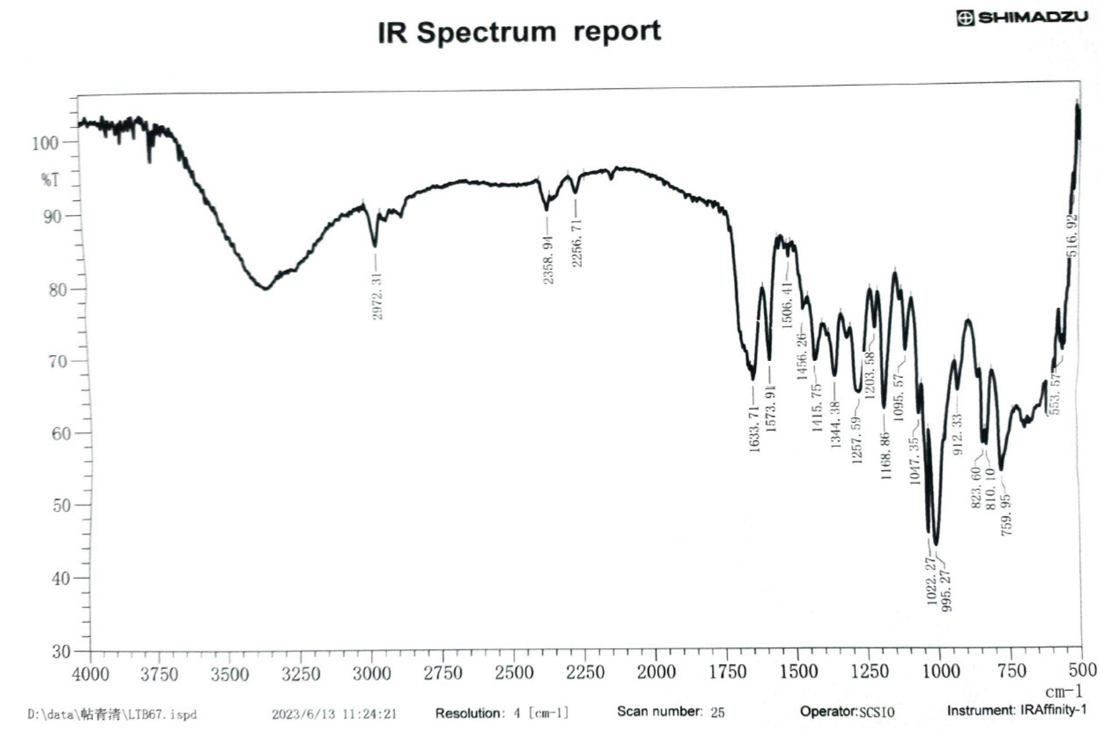

Figure S38. IR spectrum of **7**

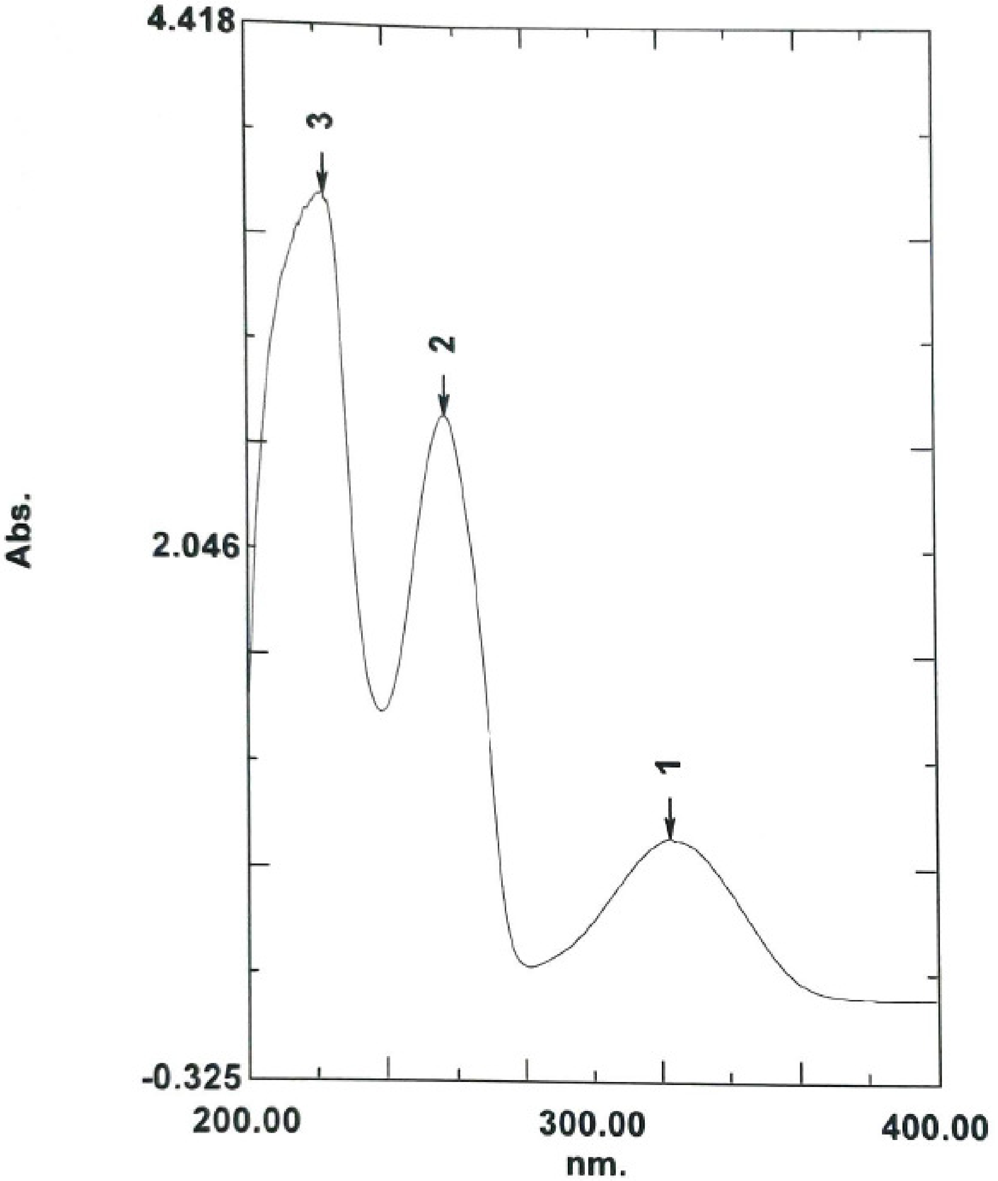

Wavelenth (nm)

No.	P/V	波长(nm)	Abs.	描述
1		322.60	0.757	
2		256.80	2.656	
3		222.20	3.658	

Figure S39. UV spectrum of **7**

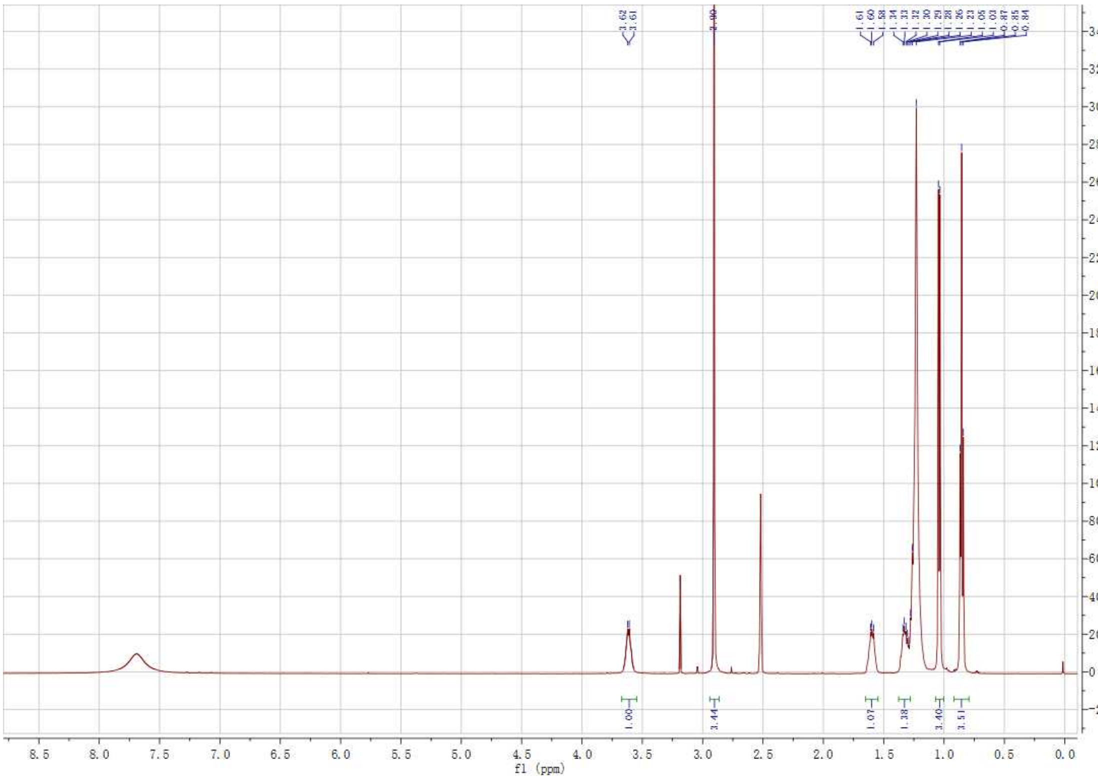

Figure S40. ^{1}H NMR spectrum of **12** (DMSO-d_6, 500 MHz)

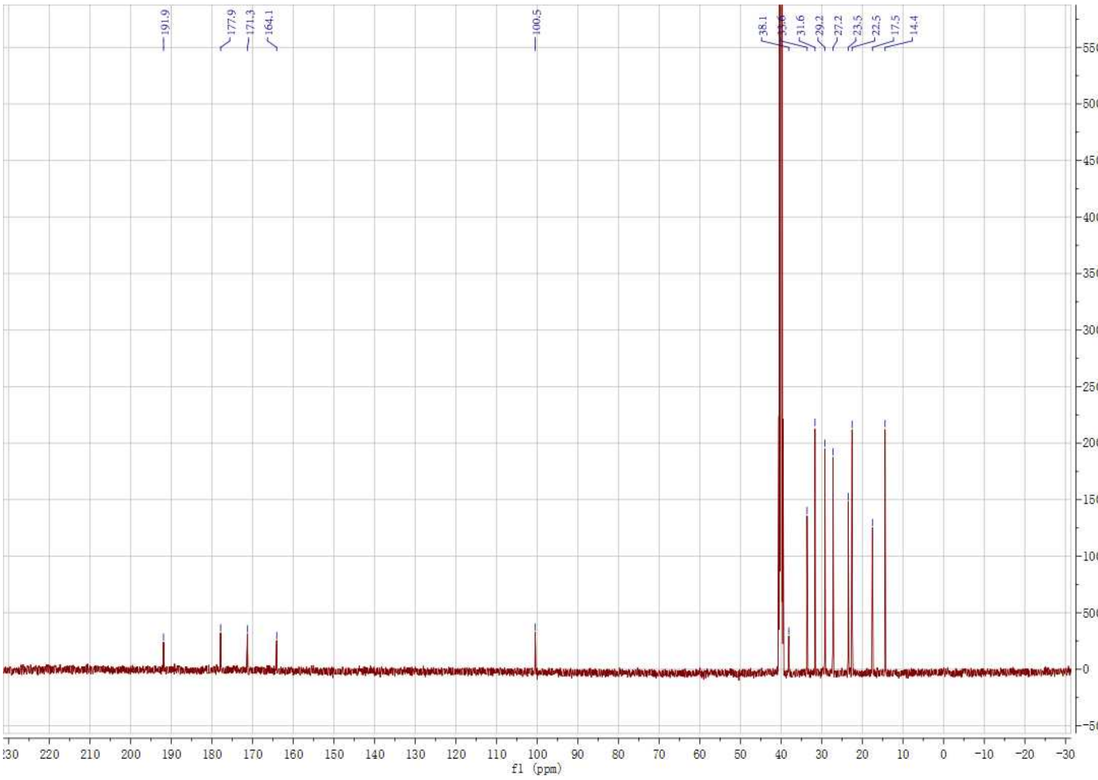

Figure S41. ^{13}C NMR spectrum of **12** (DMSO-d_6, 125 MHz)

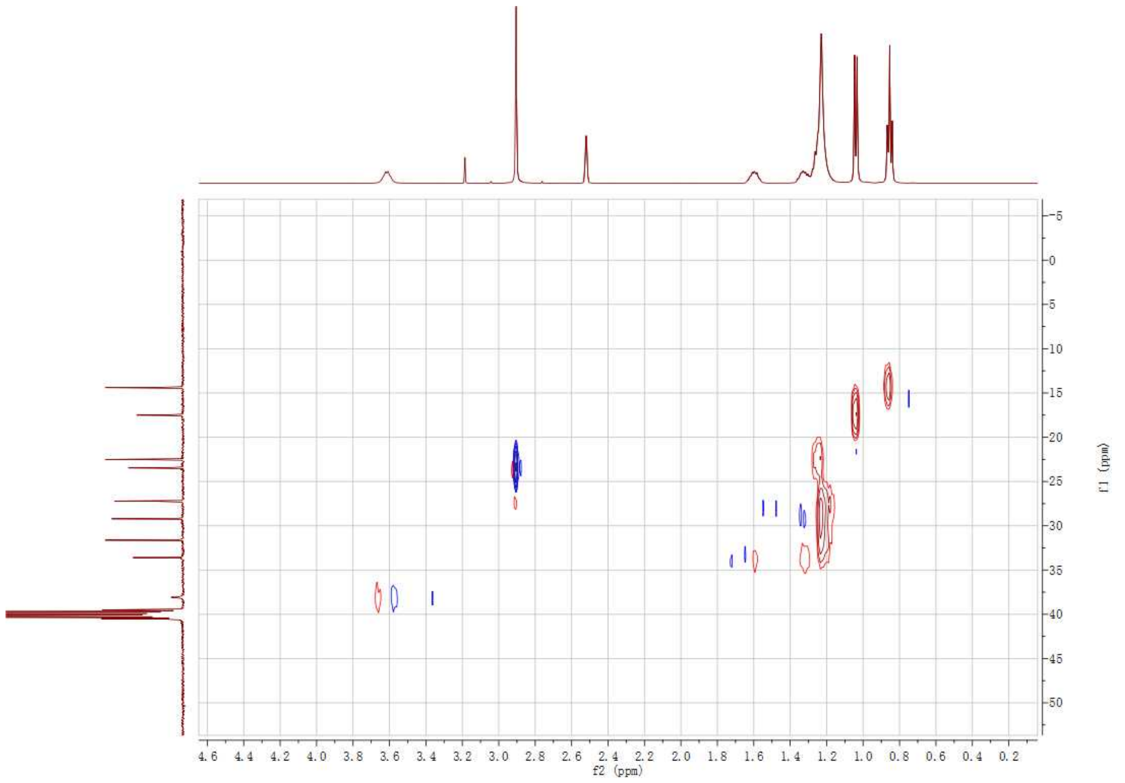

Figure S42. HSQC spectrum of **12** (DMSO-d_6)

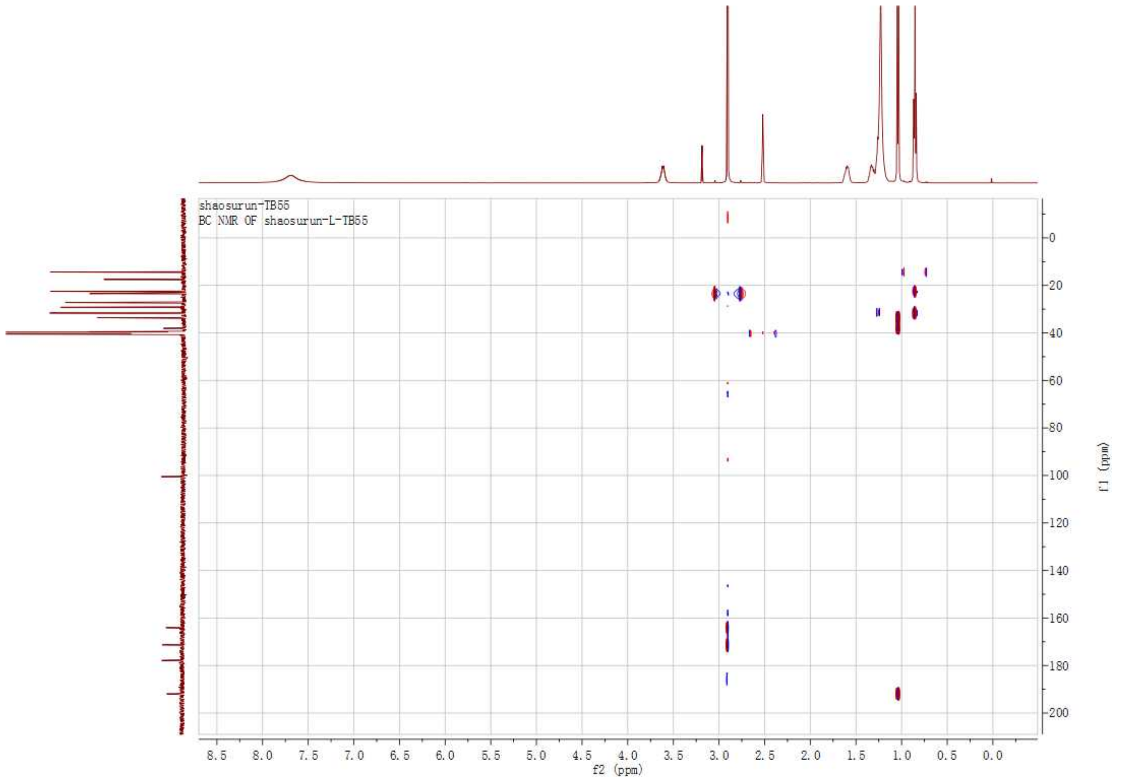

Figure S43. HMBC spectrum of **12** (DMSO-d_6)

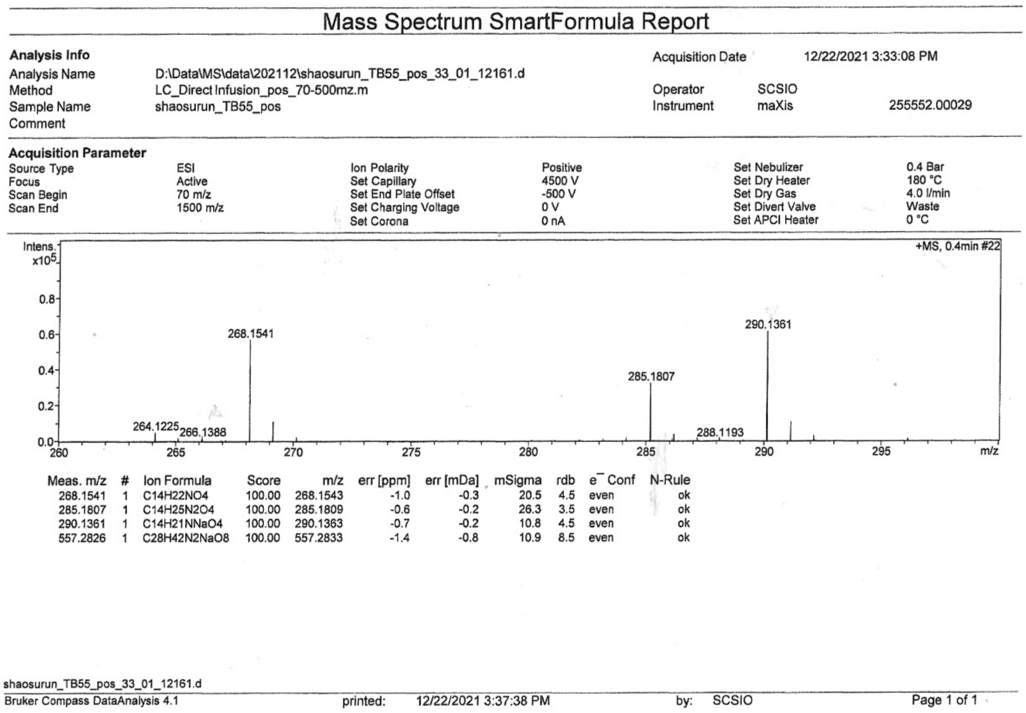

Figure S44. HRESIMS spectrum of **12**

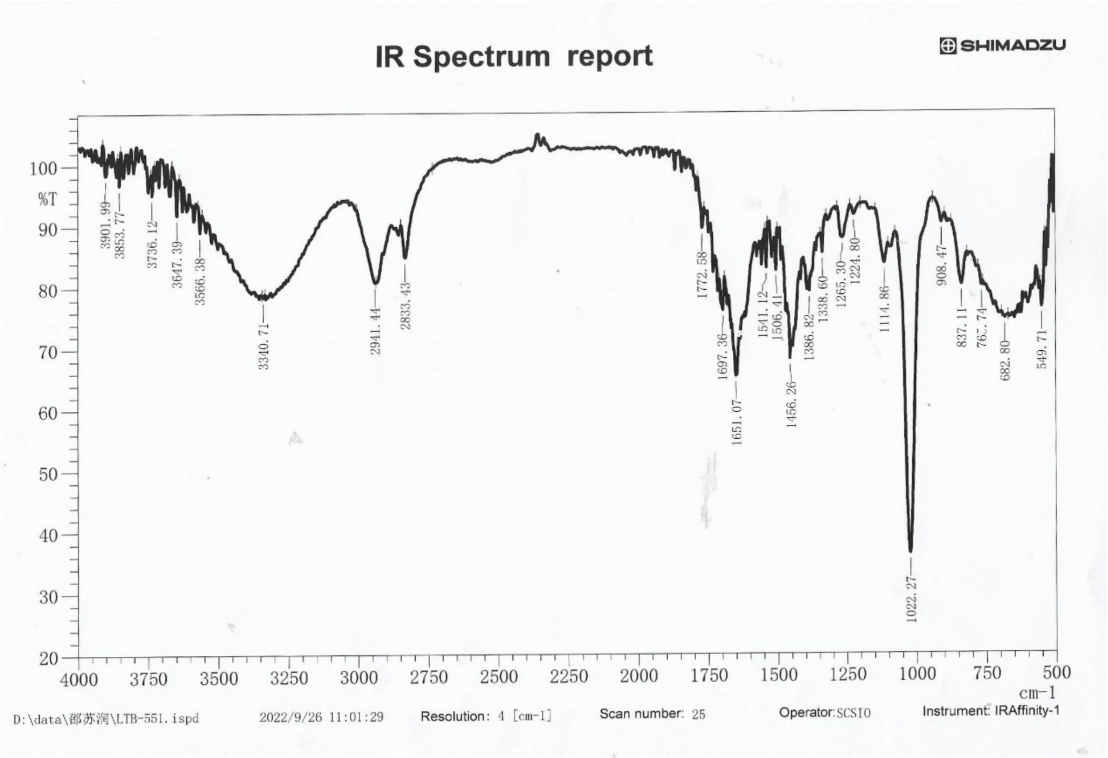

Figure S45. IR spectrum of **12**

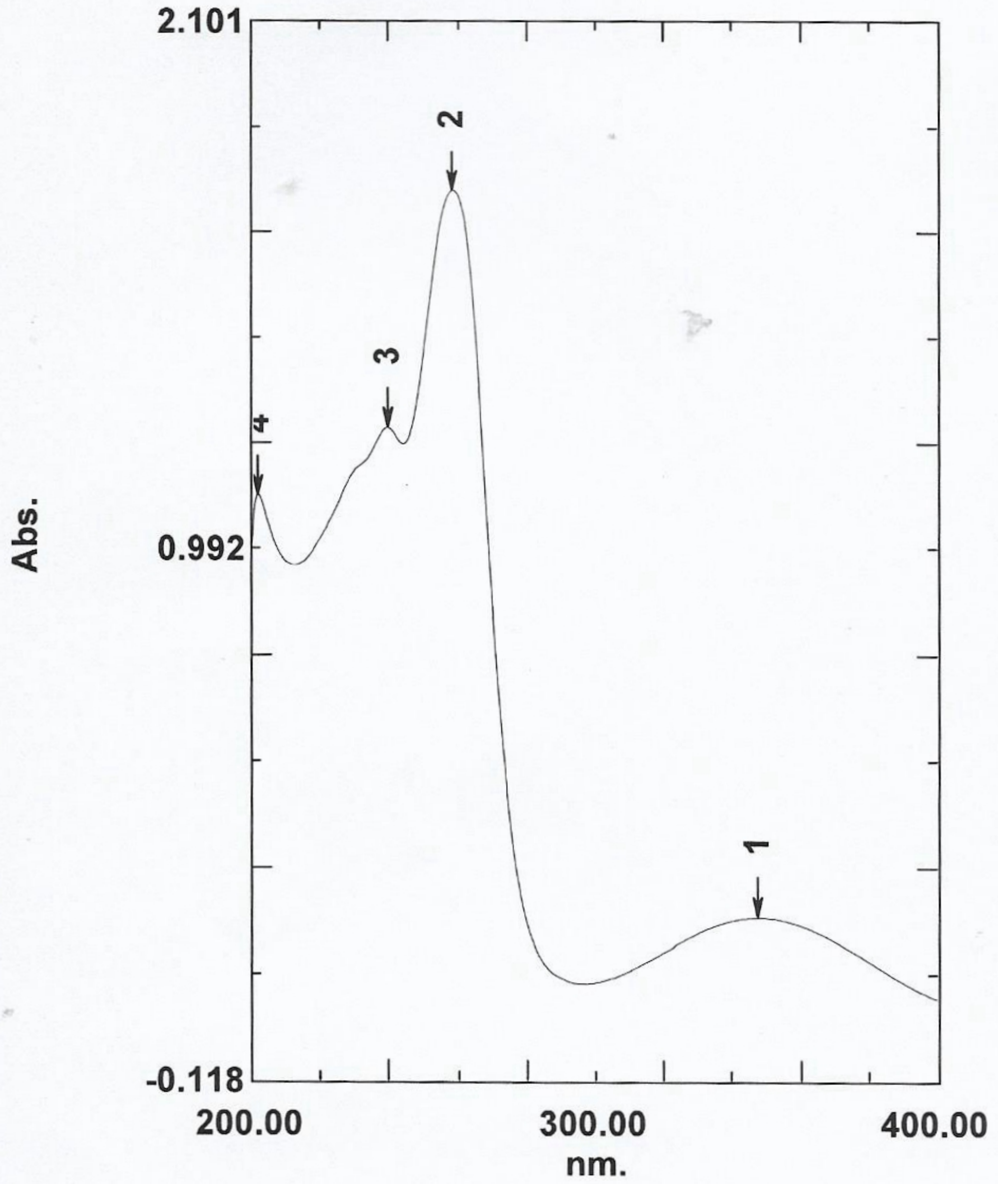

Wavelenth (nm)

No.	P/V	波长(nm)	Abs.	描述
1	⬆	347. 20	0. 223	
2	⬆	258. 60	1. 746	
3	⬆	239. 60	1. 245	
4	⬆	202. 20	1. 105	

Figure S46. UV spectrum of **12**

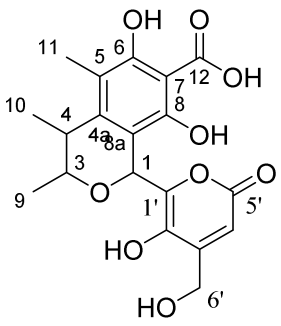

5a

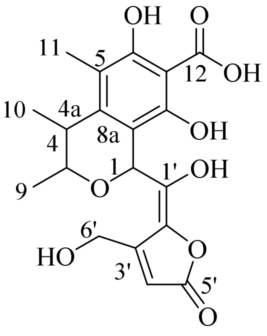

5b

Figure S47. The NMR calculations of two candidate structures (**5**a and **5**b)

5a

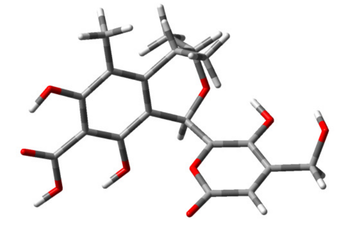	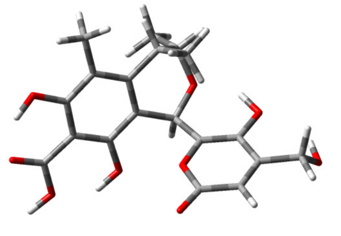	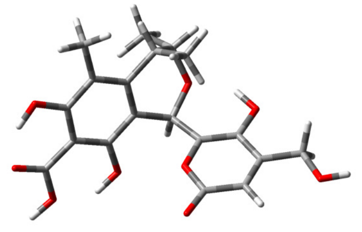
Conf.1 (7.6%)	Conf.2 (6.2%)	Conf.3 (2.0%)
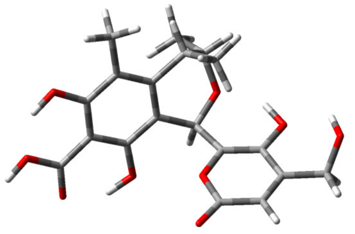	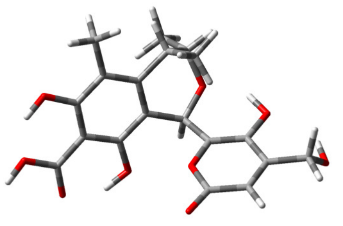	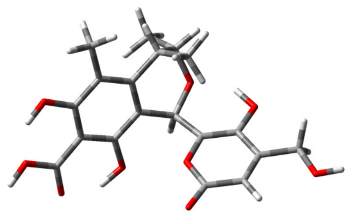
Conf.4 (6.2%)	Conf.5 (5.1%)	Conf.6 (1.7%)
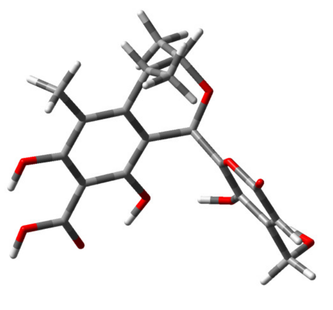	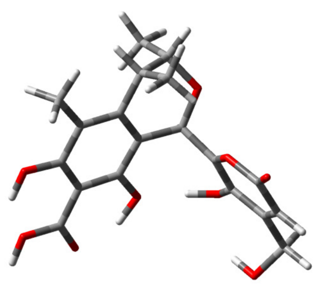	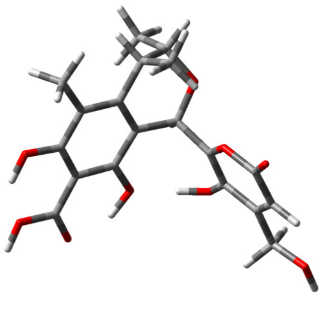
Conf.7 (17.1%)	Conf.8 (18.8%)	Conf.9 (5.3%)
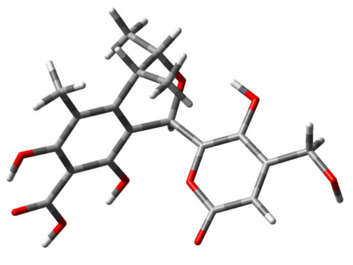	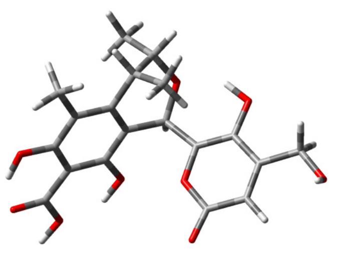	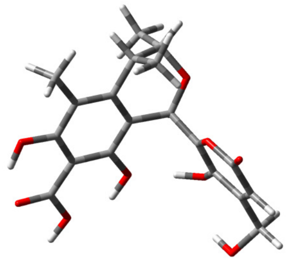
Conf.10 (3.5%)	Conf.11 (3.4%)	Conf.12 (9.5%)
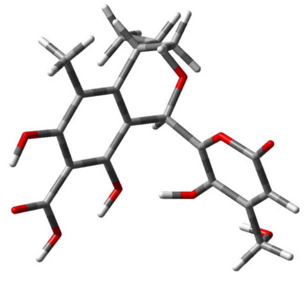	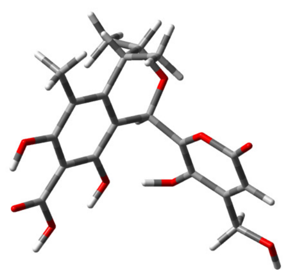	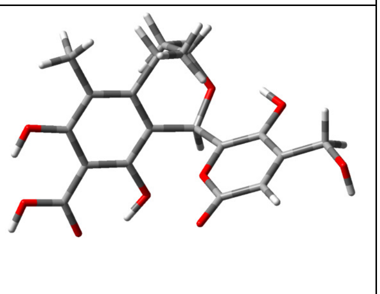
Conf.13 (7.8%)	Conf.14 (2.6%)	Conf.15 (2.9%)

5b

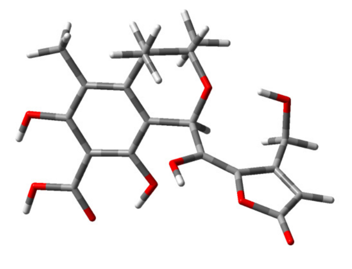	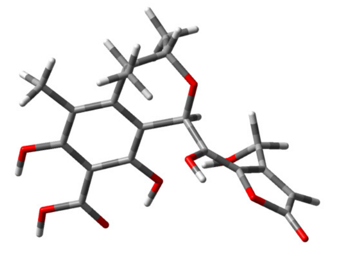	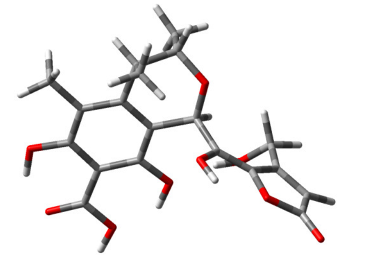
Conf.1 (25.3%)	Conf.2 (3.5%)	Conf.3 (2.1%)
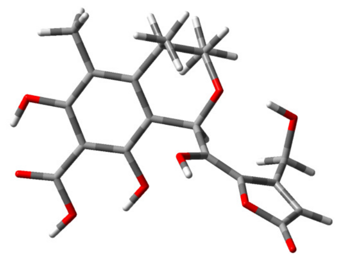	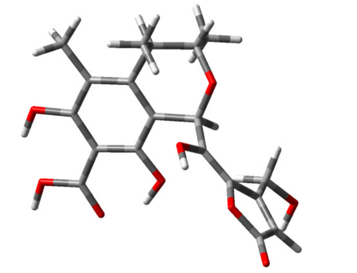	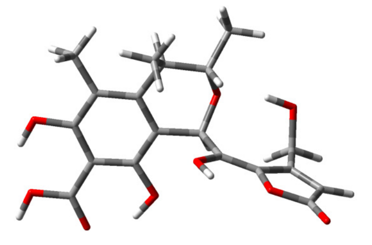
Conf.4 (31.1%)	Conf.11 (5.8%)	Conf.12 (13.9%)
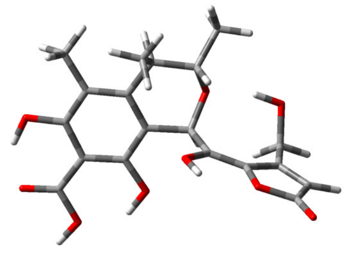		
Conf.13 (17.0%)		

Figure S48. The optimized conformers and equilibrium populations of **5**a and **5**b

Table S1. Energies of **5** at MMFF94 force field

Configuration	Conformer	Energy (kJ/mol)	Population (%)
**5a**	1	245.12	18.1
**5a**	2	245.29	16.9
**5a**	3	245.71	14.3
**5a**	4	246.47	10.5
**5a**	5	246.92	8.8
**5a**	6	247.46	7.1
**5a**	7	249.52	3.1
**5a**	8	249.69	2.9
**5a**	9	250.03	2.5
**5a**	10	250.41	2.1
**5a**	11	250.55	2.0
**5a**	12	250.61	2.0
**5a**	13	251.11	1.6
**5a**	14	251.56	1.3
**5a**	15	251.83	1.2
**5b**	1	270.60	18.8
**5b**	2	270.77	17.5
**5b**	3	270.85	17.0
**5b**	4	271.10	15.4
**5b**	5	272.58	8.4
**5b**	6	273.03	7.1
**5b**	7	274.88	3.3
**5b**	8	275.42	2.7
**5b**	9	276.76	1.6
**5b**	10	276.93	1.5
**5b**	11	277.49	1.2
**5b**	12	277.65	1.1
**5b**	13	278.25	0.9

Table S2. Energies of **5** at B3LYP/6–31+G(d, p) level in methanol

Configuration	Conformer	E (Hartree)	E (kcal/mol)	Population (%)
**5a**	1	-1413.2151505	-886806.639090255	7.6
**5a**	2	-1413.2149512	-886806.514027512	6.2
**5a**	3	-1413.2139306	-886805.873590806	2.0
**5a**	4	-1413.2149615	-886806.520490865	6.2
**5a**	5	-1413.2147744	-886806.403083744	5.1
**5a**	6	-1413.2137505	-886805.760576255	1.7
**5a**	7	-1413.2159133	-886807.117754883	17.1
**5a**	8	-1413.2160064	-886807.176176064	18.9
**5a**	9	-1413.2148047	-886806.422097297	5.3
**5a**	10	-1413.2144186	-886806.179815686	3.5
**5a**	11	-1413.2144315	-886806.187910565	3.4
**5a**	12	-1413.2153618	-886806.771683118	9.5
**5a**	13	-1413.2151724	-886806.652832724	7.8
**5a**	14	-1413.2141327	-886806.000410577	2.6
**5a**	15	-1413.2142439	-886806.070189689	2.9
**5b**	1	-1413.21398	-886805.9045898	25.3
**5b**	2	-1413.2121165	-886804.735224915	3.5
**5b**	3	-1413.2116154	-886804.420779654	2.1
**5b**	4	-1413.2141763	-886806.027770013	31.1
**5b**	5	-1413.2088589	-886802.691048339	0.1
**5b**	6	-1413.2087708	-886802.635764708	0.1
**5b**	7	-1413.2096562	-886803.191362062	0.2
**5b**	8	-1413.2100148	-886803.416387148	0.4
**5b**	9	-1413.2094884	-886803.086065884	0.2
**5b**	10	-1413.209393	-886803.02620143	0.2
**5b**	11	-1413.2093962	-886803.028209462	5.8
**5b**	12	-1413.2134105	-886805.547222855	13.9
**5b**	13	-1413.2136055	-886805.669587305	17.0

Table S3. DP4+ analysis of calculated ^{1}H & ^{13}C NMR data of **5a** and **5b** (experimental for **5**, isomers **1** and **2** for **5a** and **5b**, respectively)

A	B	C	D	E	F	G	H
Functional		Solvent?		Basis Set		Type of Data	
mPW1PW91		PCM		6-311G(d,p)		Unscaled Shifts	
		DP4+	0.02%	99.98%	-	-	-
Nuclei	sp2?	Experimental	Isomer 1	Isomer 2	Isomer 3	Isomer 4	Isomer 5
C		64.4	69.8	71.5			
C		73.3	79.6	79.1			
C		35.3	41.0	40.8			
C	x	141	156.9	157.5			
C	x	110.1	123.0	122.0			
C	x	159.4	166.6	167.1			
C	x	101.9	100.5	100.0			
C	x	156.5	162.5	162.0			
C	x	107.3	115.4	114.6			
C		18.8	22.1	20.3			
C		20	19.57	20.25			
C		10.3	12.07	12.73			
C		176	177.51	177.48			
C	x	150.1	156.65	154.52			
C	x	143.2	144.47	140.94			
C	x	167.5	163.56	166.36			
C	x	109	115.78	118.55			
C	x	174.7	168.95	176.16			
C		59.9	66.93	61.89			
H		5.88	6.26	5.99			
H		3.93	4.28	3.97			
H		2.63	2.88	2.84			
H		1.13	1.09	1.30			
H		1.26	1.50	1.23			
H		1.97	2.20	2.00			
H	x	6.26	6.17	6			
H		4.12	4.67	4.58			

A	B	C	D	E	F	G	H
Functional		Solvent?		Basis Set		Type of Data	
mPW1PW91		PCM		6-311G(d,p)		Unscaled Shifts	
		Isomer 1	Isomer 2	Isomer 3	Isomer 4	Isomer 5	Isomer 6
sDP4+ (H data)		52.38%	47.62%	-	-	-	-
sDP4+ (C data)		2.77%	97.23%	-	-	-	-
sDP4+ (all data)		3.04%	96.96%	-	-	-	-
uDP4+ (H data)		0.69%	99.31%	-	-	-	-
uDP4+ (C data)		47.35%	52.65%	-	-	-	-
uDP4+ (all data)		0.62%	99.38%	-	-	-	-
DP4+ (H data)		0.75%	99.25%	-	-	-	-
DP4+ (C data)		2.50%	97.50%	-	-	-	-
DP4+ (all data)		0.02%	99.98%	-	-	-	-
